# String Quartet No. 1 “Polar Energy Budget” – Music composition using Earth observation data of polar regions

**DOI:** 10.1016/j.isci.2024.109622

**Published:** 2024-04-18

**Authors:** Hiroto Nagai

**Affiliations:** 1 Department of Geography, Faculty of Geo-environmental Science, Rissho University, Kumagaya, Japan

## Abstract

In recent years, advancements in digital processing have facilitated the transformation of data into sound, a process referred to as *sonification*. To raise awareness and understanding of climate change, various sonification endeavors utilizing Earth science data have surfaced; nevertheless, the outcomes frequently deviate from conventional music compositions. This backstory aims to examine the possibilities and limitations of sonification by composing music based on Earth observation data with intentional and staged intervention of arrangements by a composer, presenting the music composition results and presenting the feedback and discussions raised by the audience.

**Figure:**
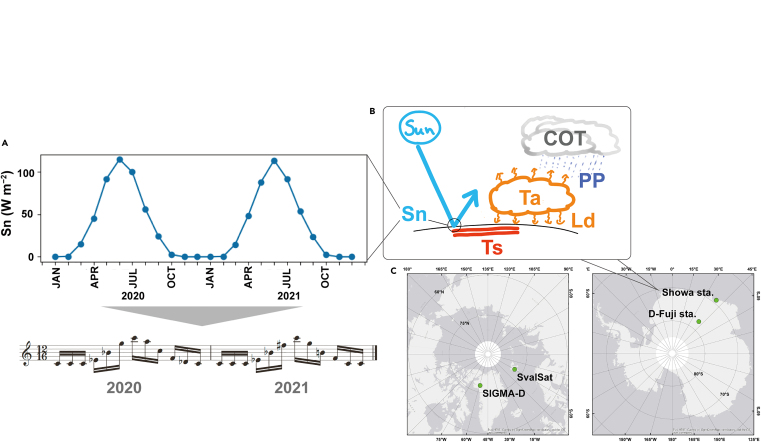
Above image: Concept illustrations (A) Basics of physical-quantity conversion to the sound. (B) Simplified energy budget system and corresponding quantities considered in this study, and (C) location of the four sites for data sampling.

In the contemporary era, where global environmental awareness is paramount, this work can be positioned as a musical composition aligned with such environmental consciousness.

The contrast between the nature-derived fluctuating periodicity under 12-tone technique and the human-derived well-designed classical harmonic progressions successfully expands the range of tension-and-relaxation expression, which has never been highlighted in the history of music. It can also be described as the endeavor of translating the “sound” of the Earth into the “song” of humanity.

## Main text

### Beginnings

Sonification is the technique of rendering sound in response to data.^1^ In the field of Earth science there have been several data sonification attempts, as introduced by Alexandra Supper.^2^ In recent years, there has been particular attention for creating music pieces which aims to raise awareness regarding climate and environmental change. Daniel Crawford composed “A Song of Our Warming Planet” and “Planetary Bands, Warming World” in which notations are generated from changes of atmospheric temperature over the past 133 years.^3^ Lee de Mora composed “Earth System Allegro” and following pieces in which notations are generated from multiple physical quantities derived from a global climate model.^4^

When a natural scientist, who is also a music enthusiast, composes through sonification, there is a tendency to avoid intentional interventions or edits (i.e., contamination) in the original data. As a result, while the information from the original data are preserved as much as possible, composed musical pieces often contain a monotonous progression and lack any significant dynamics. This may be because those pieces have the primary purpose to increase and gather public attention to the activities themselves as well as ongoing environmental changes, not by means of general graphic presentations, but by means of sound. In this case, the evolution from sound (without evoking emotions in listeners) to music (intentionally designed to affect listeners’ emotions) is not necessary. While these may be considered as one of the characteristics of such works, the composers still lack means and perspectives to intentionally arrange the music based on their liberal and unique ideas.

As a fundamental principle in music composition, it is necessary to combine temporal sequences from tension-building to resolution in various scales, from harmonic progressions to entire movements. It is necessary to actively intervene and affect the emotions of the audience.

So far, there have not been any published attempts or open discussion on sonification-based music composition, nor attempts to demonstrate the methodology required to intentionally affect the audience’s emotions with an artistic piece. This backstory, therefore, aims to highlight the possibilities and challenges of using sonification alongside traditional music compositions. By converting the temporal changes in Earth observation data into sound data, and gradually introducing intentional musical stylistic interventions, a piece for a string quartet was produced. The composition includes both non-human–derived melodies as well as the composer’s free arrangements. The work was shared with the public, and opinions from various perspectives were gathered to discuss the future directions of data sonification and their contribution to science communication and outreach, as well as their unprecedented benefits to the field of art.

## Art and science

The author and composer has titled the new work *String Quartet No. 1 “Polar Energy Budget”.* The term “energy budget” refers to the input and output of physical energy on the Earth’s surface originating from solar radiation. This energy circulates through the land, atmosphere, and oceans, driving the Earth system as it changes physical forms^5^^,^^6^. The initial concept for this work included eliciting a sense of diverse imagination and narrative from the invisible exchange of energy occurring at the surface of Earth’s polar regions.

### Which Earth observation data were used to produce the composition?

Publicly available data on shortwave net radiation (Sn), downward longwave radiation (Ld), and precipitation (PP) are obtained from the land component of the fifth generation of European ReAnalysis (ERA5), provided by the Copernicus Climate Change Service of the European Commission and the European Center for Medium-Range Weather Forecasts.^7^ An advanced dataset, ERA5-Land,^8^ was used in this study, of which spatial resolution improved from original 31 km–9 km.^9^ Recorded data of surface temperature (Ts)^10^ and cloud optical thickness (COT)^11^ are obtained from MODIS (Moderate Resolution Imaging Spectroradiometer) datasets.

Related parameters are employed, with which simplified energy-budget system can be drawn. Initially, the shortwave net radiation raises the Earth’s surface temperature. It warms the atmosphere, resulting in fluctuations of atmospheric temperature and downward longwave radiation. The thermal energy within the land and atmosphere contributes to cloud formation and progression, influencing cloud optical thickness. Well-developed clouds, in turn, lead to precipitation, a fundamental element for the global water cycle, ecology, and geomorphological processes.

### Sampling locations

This study has four points in polar regions for data sampling (supplemental file S1). The SIGMA-D in the Greenland Ice Sheet has extremely cold air-temperature variations mostly below 0°C. The Svalbard Satellite Station (SvalSat) is a facility for satellite operation in Spitzbergen, the Svalbard archipelago in Arctic Norway. It has a tundra climate with the air-temperature variations of approximately −20 to +5°C. Showa Station (Showa sta.) is a Japanese research base located in Antarctica. The air temperature exhibits a seasonal cycle, reaching maximums around 5°C and minimums around −30°C. Dome Fuji Station (D-Fuji sta.) is a Japanese research facility in inland Antarctica, characterized by less precipitation (<50 mm year^−1^) and extremely low temperatures (approximately −80 °C at minimum).

### Which musical instruments were selected for this work?

In this study we selected a string quartet, comprising two violins, a viola, and a cello. This choice was based on the fact that string quartets have the traditional four-voice structure (i.e., soprano, alto, tenor, and bass). Another reason is the diversity of playing techniques of bowed instruments. In addition to the common arco technique (i.e., bowing the four strings by an arco), pizzicato (pizz.: plucking the strings with the fingers) creates an entirely different ‘bounced’ impression. Moreover, nuanced variations in playing techniques, such as producing a harsh sound (sul ponticello) or a soft sound (sul tasto), can be achieved by adjusting the distance of the bow from the bridge. These techniques, frequently seen in classic to popular music, were used to add diverse nuances and expressions to the music based on the composer’s creative imagination. These arrangements are performed independently from the types of physical quantity and ranges of dynamics in the input data.

### How was the Earth observation data turned into music?

The initial processing involves extracting one-dimensional temporal profiles of the physical quantities described above (Sd, Ld, PP, Ts, COT) since 1982 at the four target sites using a browser-based code editor of Google Earth Engine (GEE) - an online platform which freely enables access to peta-byte-scale satellite data and other geo-spatial dataset.^12^ The output CSV files are then converted into Musical Instruments Digital Interface (MIDI) format using Mido, a Python module developed by Ole Martin Bjørndalen, Raphaël Doursenaud, and other contributors (https://mido.readthedocs.io/en/stable/). Users must define the essential parameters including the basic duration of musical notes, the range, and the lowest pitch for each instrument (supplemental file S2). For this study, all 12 tones comprising the chromatic scale are allowed, and no specific scales are defined. This is one of the forms of contemporary music known as “12-tone technique.” Referring to the maximum and minimum values, all data in a file is normalized to span two, three, or four octaves.

Extracted MIDI files are imported to a commercial software of Digital Audio Workstation (DAW), Logic Pro (Apple Inc.). On its interface, the user can freely shift the pitch of the entire passage and can quickly experiment with cuts and movements in the direction of measures while listening to the automated playback. It is challenging to flexibly alter the range of pitch within the DAW. Changing the range of pitches results in intervals that do not harmonize with other parts. It is therefore necessary to pre-generate multiple patterns of the range through Python parameter settings and choose the optimal one.

In this process, various musical arrangements are executed as below.

(1) Manipulating initial note and pitch range
(2) Obtaining melodic sequences and harmonic structures from the numerical sequence of solar constant (1.366 kW m^−2^)
(3) Changing the ensemble format, either reducing or increasing instruments for play
(4) Overlaying passages created from different data
(5) Adopting various playing techniques such as tremoro, stacato, pizz., sul tasto, and sul ponticello
(6) Forming rhythms by eliminating some of originally present notes
(7) Generating a contrast between tension and relaxation through dynamics (forte or piano)
(8) Incorporating chord progressions and melodies based on the traditional rule of harmony (i.e., functional harmony)
(9) Combining handwritten parts with data-derived passages
(10) Gradually transitioning between two ensembles through changes in volume

The resulting piece was notated with detailed playing techniques by means of a music notation software, Finale 27 (MakeMusic Inc.). It was then printed as sheet music and distributed to the performers to be played (supplemental file S3).

## Introducing *String Quartet No. 1 “Polar Energy Budget”*

*String Quartet No. 1 “Polar Energy Budget”* is a chamber music composition, with a duration of approximately 6 min with a full score ranging 8 pages containing 148 measures (supplemental file S3). Recoded and edited performance (Video S1) was uploaded to YouTube.^13^^,^^14^ The detailed structure of the composition are described below, in which each [symbols] corresponds to rehearsal numbers marked in the score.

Video S1. Polar Energy Budget

The [Intro.] section is composed of almost unaltered output values of ERA5-derived net solar radiation (Sn) data without intentional music editing. Sequential monthly-averaged values since 1981 are performed. One measure (i.e., musical segment defined by a given number of beats between two bars) contains 12 tones, which represents a one-year range. Through several times of trial and error, two-octave range for MIDI output contributes for the most natural and playable score. Smaller ranges cause continuous same tones, whereas larger ranges make it difficult to play fluently with many distant “jumping” pitches.

The performance starting from the cello (as D-Fuji sta.) gradually adding other instruments from lower parts to the Violin 1st (as SIGMA-D), creating a dense-and-tense atmosphere. Respective lowest notes have been set to C-E-A-A. This is a representation where the sequence numbers of the solar constant (1.366 kW m^−2^) are converted into a sequence of musical pitch (1-3-6-6 to C-E-A-A). Here, a measure starts with a note just before the seasonal lowest (0 W m^−2^), which forms minimal descending scales, successfully causing a sense of progression and dynamism.

The cello and viola perform ascending and descending passages from the third to the fourth beat, while the two violin parts, joining in midway, execute ascending and descending movements from the first to the second beat. This symbolism reflects the reversal of seasons between the southern and northern hemispheres, where sunlight intensity increases. The sustained lowest note indicates the absence of sunlight during the polar night when solar radiation is 0 W m^−2^.

In Section [A], melodies converted from 8-day composites of surface temperature (Ts) data obtained from MODIS is inserted into the second violin and viola parts. The rhythm does not synchronize with other parts because of the different phases (8 days versus 30 days for one note), resulting in an independent motion. Unlike Sn, Ts generally follows a sinusoidal pattern, but the maximum and minimum temperatures vary from year to year. From the 37th measure, the composer edited repeating measures with crescendo (i.e., gradual volume increase), allowing for a clear transition to the next section.

Sections [B] and [C] are constructed from longwave downward radiation (Ld) values, with two measures equivalent to one year of data. The first violin takes on a solo role first (i.e., playing melody alone), played with a bowing technique, while the other parts plays rhythms with pizz. This rhythm is created by removing unnecessary notes. From the 73rd measure, expression as piano with sul ponticello (i.e., bowing the strings as close to the forward “bridge” as possible) produces a raspy sound, expressing a sense of restlessness.

Section [D] begins with cascaded tense passages from high to low parts using tremors (i.e., bowing with small, rapid up-and-down movements), and fortissimo (i.e., significantly large volume), creating a harsh and unyielding atmosphere. This section emulates one of the typical styles by a Russian composer, Dmitri Shostakovich. The fundamental motif (i.e., minimal unit for melody construction) is a snippet taken from [C]. It converges into a chord composed of C-E-A-A, originated from the sequence numbers of the solar constant.

In Section [E], no sonification is used. The C-E-A-A harmony transitions through an Amen cadence (F-major to C-major harmony), which is a traditional ending frequently seen in Christian hymns. In Section [F], a transposed motif of the solar constant, G-B-E-E is expanded by viola as a warm and expressive solo supported by a classical style of harmony. It gives an effect of anticipation and reassurance for the listener, in contrast to the 12-tone techniques used before this section.

In Section [G], a passage derived from the MODIS Cloud Optical Thickness (COT) data is assigned. The cello plays a simple bass rhythm alone, which gradually shifts into a brisker pace, reminiscent of Paul Dukas’s “The Sorcerer’s Apprentice,” aiming to build a sense of anticipation leading to the climax. Both sonification-derived passages and artificial rhythms are included in this section.

In Section [H], the viola plays artificial melody developed from the G-B-E-E motif, while the cello performs a simple bass progression. From the 125th measure, the two violins depict the monthly variation in precipitation (PP). Relatively larger-terms of diminuendos (i.e., gradually decreasing volume) and crescendos (i.e., gradually increasing volume) are given to these two groups. The resulting feeling is that the handwritten sound is being invaded by the irregular-pulse sound of nature, evoking thoughts of a conflict between humans and nature.

Section [I] serves as the finale. It contains precipitation (PP)-derived passages, in which little periodicity is observed, creating a sequence of sounds without regularity. In the realm of previous contemporary music, creating such sequences required mathematical approaches such as using random numbers. However, a novel approach has emerged here, extracting sequences from the natural world.

### How was this piece presented to the public?

The recording and footage of the performance by a Japanese professional string quartet, PRT Quartet, were edited into a single video clip using Adobe Premiere Pro (Adobe Inc.). The recording was then released on YouTube in March 2023.^13^ Another version with visual commentary was also published (Video S1).^14^

Simultaneously, a “premiere” workshop was held at Waseda University in Tokyo on March 18, 2023.^15^ A keynote lecture provided an overview of sonification using scientific data, a presentation of the composition, and an explanation of the composer’s intent. This was followed by a panel discussion with three earth-remote-sensing experts and an undergraduate student studying remote sensing. They all have significant amateur experiences and skills in music and art. The key parts of the transcript are archived as supplemental file S4.

### What was the audience’s reaction to the music, and what reflections can be drawn from the panel discussion?

Several comments and opinions given by the panelists (supplemental file S4) are discussed and evaluated here. One of the main insights from the participants is that music, unlike usual graphical representations of scientific data, evokes emotional impression first, which is soon followed by their interest or curiosity in the scientific data. It grabs the audiences’ attention forcefully, while graphical representations require active and conscious recognition instead. This reveals the potential for outreach in the Earth sciences through music. One significant feature is that the completeness of seasonal periodicity shown in each data-derived passage is hindered by irregular fluctuations, and the intensity of the fluctuation varies among physical quantities. For example, at the beginning of the music, the cello plays seasonal patterns of monthly solar radiation. It does not perfectly repeat the same passage but presents slightly different passages year by year. These variations represent basic seasonal patterns and possible ranges of uncertainties. On the other hand, toward the end of the music, the monthly precipitation amount does not exhibit remarkable seasonal periodicity but rather demonstrates an almost random pattern. With consideration of such differences among physical quantities, it might also be possible to detect anomalous weather patterns audibly. Anomalies in climate patterns would produce sounds deviating from the norm, acting as a source of insight.

### Challenges and opportunities

#### Perspectives on art and science relationships

In the discussion, one panelist mentioned about the intensity of composer’s manipulation to be a music piece. The composition process and consequent appreciation reveals that sonification music and characteristics of the source data are in an independent relationship. Sound data from specific natural phenomena does not automatically evoke some kinds of impression associated with that phenomenon. For example, melodies derived from solar radiation do not inherently evoke the shining sun, nor do the melodies derived from temperature rise evoke a sense of warmth. Conversely, composers are not obligated to connect objective natural data with subjective emotions. They are free to use them merely as components in a work. This can be considered a significant reason why conventional sonification works have struggled to establish a firm artistic status. Many of them have garnered attention solely for being sonification, failing to unfold further expressive depth. Basic knowledge of musical theory and/or collaborations between artist and scientist, is therefore needed to intentionally design what the composer wishes to express.

If music with sonification is used for storytelling in the field of Earth sciences, providing information that the composition is based on Earth observations before the audience experiences it can enhance their attention and contribute to a more emotionally stirring experience. Even in this case, it is essential to translate the desired scenes and emotions into resonances using a methodology aligned with conventional musical syntax. As seen in most of classic and even modern commercial music, dramatically portraying cycles of tension and relaxation across various scales, from note units to section units, is recognized as an effective means of influencing the emotions of the audience. This study successfully highlights the contrast between the nature-derived fluctuating periodicity under 12-tone technique and the human-derived well-designed classical harmonic progressions successfully expands the range of tension-and-relaxation expression, which has never been highlighted in the history of music. It can also be described as the endeavor of translating the “sound” of the Earth into the “song” of humanity.

### Conclusions

This project successfully presents a composition through sonification that aligns more closely with traditional music. The potential for storytelling extends beyond geographic spatial information (i.e., remote-sensing data) to encompass various scientific data fields. For example, one could create a percussion ensemble based on the seismic history in different regions or compose a grand symphony portraying Earth’s history using proxy data extracted from ice cores and lake sediment.

Music has evolved throughout different eras, adapting to the historical and political contexts of each period, from the time of J.S. Bach. Meanwhile, cloud processing technology has made vast amounts of Earth science data accessible to anyone. This marks a significant turning point from an era where only scientists handled data to an era where artists can freely use data to create their works. By establishing optimized platforms and web tools, it is possible to provide Earth science data as new material to various artists. In the contemporary era, where global environmental awareness is paramount, this work can be positioned as a musical composition aligned with environmental consciousness.

## Figures and Tables

**Figure:**
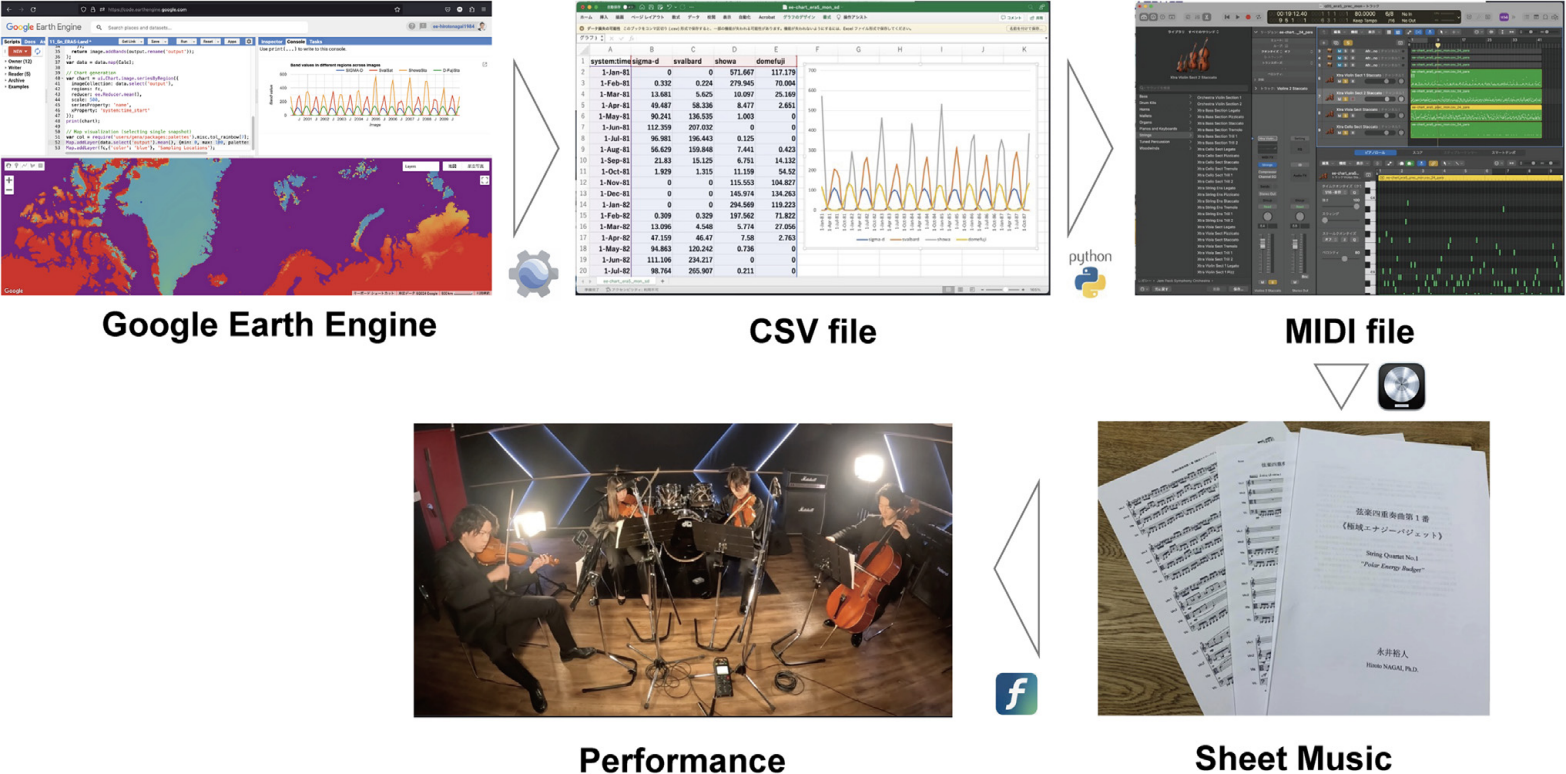
Flow of composition

**Table:** Source of physical quantities used in this study

Physical quantity	Abbreviat ion	Unit	Spatial resolution (km)	Temporal step	Data source	Directory path in GEE	Quantity name in GEE
Downward solar radiation	Sd	W m^−2^	11.1 km	monthly	ERA5-Land	ECMWF/ERA5_LAND/MONTHLY	surface_net_solar_radiation
Surface temperature	Ts	K	1.0 km	8-day	MODIS	MODIS/006/MOD11A2	LST_Day_1km
Atmospheric temperature (to Ld)	Ta	K	11.1 km	monthly	ERA5-Land	ECMWF/ERA5_LAND/MONTHLY	temperature_2m
Cloud Optical Thickness	COT	n.a	11.1 km	monthly	MODIS	MODIS/061/MOD08_M3	Cloud_Optical_Thickness_Liquid_Log_ Mean_Mean
Precipitation	PP	mm	11.1 km	hourly	ERA5-Land	ECMWF/ERA5_LAND/HOURLY	total_precipitation

## References

[1] Hermann T., Hunt A., Neuhoff J.G., Hermann T., Hunt A., Neuhoff J.G. (2011). The Sonification Handbook.

[2] Supper A. (2012).

[3] George S.S., Crawford D., Reubold T., Giorgi E. (2017). Making Climate Data Sing: Using Music-like Sonifications to Convey a Key Climate Record. Bull. Am. Meteorol. Soc..

[4] de Mora L., Sellar A.A., Yool A., Palmieri J., Smith R.S., Kuhlbrodt T., Parker R.J., Walton J., Blackford J.C., Jones C.G. (2020). Earth system music: music generated from the United Kingdom Earth System Model (UKESM1). Geosci. Commun..

[5] Trenberth K.E., Fasullo J.T., Kiehl J. (2009). Earth's global energy budget. Bull. Am. Meteorol. Soc..

[6] National Aeronautics and Space Administration, Science Mission Directorate (2024). The Earth's Radiation Budget.

[7] Hersbach H., Bell B., Berrisford P., Hirahara S., Horányi A., Muñoz-Sabater J., Nicolas J., Peubey C., Radu R., Schepers D. (2020). The ERA5 global reanalysis. Q. J. R. Meteorol. Soc..

[8] Muñoz-Sabater J. (2019). Copernicus Climate Change Service (C3S) Climate Data Store (CDS).

[9] Muñoz-Sabater J., Dutra E., Agustí-Panareda A., Albergel C., Arduini G., Balsamo G., Boussetta S., Choulga M., Harrigan S., Hersbach H. (2021). ERA5-Land: a state-of-the-art global reanalysis dataset for land applications. Earth Syst. Sci. Data.

[10] Wan Z., Hook S., Hulley G. (2021). NASA EOSDIS Land Processes Distributed Active Archive Center.

[11] Platnick S., Hubanks P., Meyer K., King M.D. (2015).

[12] Gorelick N., Hancher M., Dixon M., Ilyushchenko S., Thau D., Moore R. (2017). Google Earth Engine: Planetary-scale geospatial analysis for everyone. Remote Sens. Environ..

[13] Nagai H. (2023). 弦楽四重奏曲第1番⟪極域エナジーバジェット⟫ (2022)/String Quartet No.1 "Polar Energy Budget" 作曲:永井裕人/Hiroto NAGAI. https://youtu.be/fRg0ax4u67o?si=Va02GDzzJaKMqceJ.

[14] Nagai H. (2023). String Quartet No.1 "Polar Energy Budget" by Hiroto NAGAI [Performance Video for Study]. https://youtu.be/Tulsx2wt3qU?si=mXHPPWnc-2fsaeZY.

[15] Nagai H. (2023). 基調講演「衛星データ可聴化技法の探究」(永井裕人)"Exploration of Sonification from Satellite Data" (by Hiroto Nagai). https://youtu.be/bChLiZTiZ9k?si=iSjc2u3x61y7oB7h.

