# Supplementary material for: String Quartet No. 1 “Polar Energy Budget” – Music composition using Earth observation data of polar regions
Source: iScience. 2024 Apr 18;27(5):109622. doi: 10.1016/j.isci.2024.109622 (PMC11123573; doi:10.1016/j.isci.2024.109622)
Supplement: Document S1. Supplemental files S1–S4 [file mmc1.pdf]

iScience, Volume *27*

Supplemental information

String Quartet No. 1 “Polar Energy Budget” – Music composition using Earth observation data of polar regions

Hiroto Nagai

Supplemental files

List of contents

S1 Detail information: four locations for data sampling sp.1
S2 S2 Sample of source code
-Code1 -GEE- sp.4
-Code2 -Python- sp.5
S3 Score sp.7
S4 Extracted script from the panel discussion sp.28

Video S1

String Quartet No.1 "Polar Energy Budget" by Hiroto NAGAI [Performance Video for Study]

S1 Detail information: four locations for data sampling

In this study, the temporal variations of multiple physical quantities at specific four locations are translated into musical pitches. The magnitude of the physical quantities is represented in the pitch, with higher values corresponding to higher pitches. Two locations from the Arctic region and two from the Antarctic region are selected and assigned to the four instruments (Table A1). Details are described below.

Table S1) Details of four sampling sites. Climate name is based on Köppen climate classification.

Site name	Latitude	Longitude	Elevation (m)	Location	Landcover	Climate	Major facilities	Assigned part
**SIGMA-D**	77.6° N	59.1° W	2100	Greenland	Ice	Ice-cap	Automatic weather station	Violin 1st.
**Svalvard satellite station (SvalSat)**	78.2° N	15.4° E	~500	Spitzbergen	Bare soil	Tundra	Satellite-communication antennas	Violin 2nd.
**Showa Station (Showa sta.)**	69.0° S	39.6° E	~30	Antarctica	Bare rock	Ice-cap	Manned base for Antarctic observation	Viola
**Dome Fuji Station (D-Fuji sta.)**	77.3° S	39.7° E	3810	Antarctica	Ice	Ice-cap	Temporary manned base for ice-core drilling (*closed)	Cello

SIGMA-D for Violin 1st.

The SIGMA-D observation site, in Greenland for glaciological and meteorological research, was established by multiple Japanese research projects.[1] Located at [77.6°N; 59.1°W, 2100 meters above sea level (m a.s.l.)], the SIGMA-D site is situated within an uninhabited area on the Greenland Ice Sheet, approximately 250 km east of Qaanaaq, the nearest major residential area along the west coast of Greenland. It features an ice-cap climate (classified as EF in the Köppen climate classification system), characterized by extremely cold air temperatures typically ranging from approximately −40 to −5°C according to monthly ERA5-Land data.[2] During winter, the site experiences polar night, with the sun not rising, while in summer, it transitions to white nights, during which the sun does not set.

SvalSat for Violin 2nd.

The Svalbard Satellite Station (SvalSat) [78.2°N; 15.4°E, ~500 m a.s.l.] is a satellite operation facility located in Spitzbergen, part of the Svalbard archipelago in Arctic Norway. Established in the 1990s, SvalSat is operated by Kongsberg Satellite Services (KSAT).[3] It comprises multiple antennas used for satellite communication, including Earth observation satellites. Data collected at the station are downlinked and transmitted to various space agencies and companies worldwide. The prevailing climate at SvalSat is tundra (classified as ET in the Köppen climate classification system), characterized by seasonal air temperature variations around 0°C, typically ranging from approximately −20 to +5°C.[2]

Showa sta. for Viola

Showa Station (Showa sta.) [39.6°E; 69.0°S, ~30 m a.s.l.] is a Japanese research base situated in Antarctica, specifically on East Ongul Island within the Lützow-Holm Bay area.[4,5] Established in 1957, it stands as Japan's permanent facility dedicated to Antarctic research, serving as a central hub for diverse scientific inquiries. Operating throughout the year, Showa Station perseveres through the severe Antarctic environment, conducting research across disciplines including glaciology, meteorology, biology, and astronomy.

Constructed upon exposed bedrock, the station's buildings experience seasonal snow cover. According to records from the Japan Meteorological Agency (https://www.data.jma.go.jp/antarctic/indexant.html), the air temperature follows a seasonal cycle, with peaks around 5°C and lows around −30°C. During winter, the coastal region surrounding Showa Station becomes isolated by thick, expansive sea ice. However, in summer, thinner sea ice allows for the accessibility of icebreakers, enabling year-round station operations. This diverse environment fosters a unique ecosystem, hosting colonies of Adélie penguins (*Pygoscelis adeliae*) and various endemic species.

D-Fuji sta. for Violoncello

Dome Fuji Station (D-Fuji sta.) [39.7°E; 77.3°S, 3810 m a.s.l.] is a Japanese research facility founded in 1995, located in the East Antarctic Plateau. Positioned approximately 1000 km inland from the coastline, it encounters minimal precipitation.[6] This station was primarily established for ice core drilling purposes, aiming to access the oldest ice layers feasible.[7] To achieve this goal, a site with high elevation within the Antarctic ice sheet, akin to the summit of the ice sheet, was chosen. The area is characterized by exceptionally frigid temperatures (reaching approximately −80°C at its coldest) and relatively low atmospheric pressure (approximately 600 hPa). No terrestrial ecology has been observed in this vicinity.

References

1. Matoba, S., Motoyama, H., Fujita, K., Yamasaki, T., Minowa, M., Onuma, Y., Komuro, Y., Aoki, T., Yamaguchi, S., Sugiyama, S., et al. (2015). Glaciological and meteorological observations at the SIGMA-D site, northwestern Greenland Ice Sheet. Bull. Glaciol. Res. 33, 7–14. 10.5331/bgr.33.7.
2. Muñoz-Sabater, J., Dutra, E., Agustí-Panareda, A., Albergel, C., Arduini, G., Balsamo, G., Boussetta, S., Choulga, M., Harrigan, S., Hersbach, H., et al. (2021). ERA5-Land: a state-of-the-art global reanalysis dataset for land applications. Earth Syst. Sci. Data 13, 4349–4383. 10.5194/essd-13-4349-2021.
3. Skatteboe, R., and Kjeldsen, A.A. (2013). Interoperability Reduces Cost and Risk at Svalbard Satellite Station. In Space OPS 2004 Conference. 10.2514/6.2004-348-188.
4. Tsuji, M., and Kudoh, S. (2020). Soil Yeasts in the Vicinity of Syowa Station, East Antarctica: Their Diversity and Extracellular Enzymes, Cold Adaptation Strategies, and Secondary Metabolites. Sustain. Sci. Pract. Policy 12(11), 4518. 10.3390/su12114518.
5. Kinase, T., Adachi, K., Oshima, N., Goto-Azuma, K., Ogawa-Tsukagawa, Y., Kondo, Y., Moteki, N., Ohata, S.,

Mori, T., Hayashi, M., et al. (2020). Concentrations and size distributions of black carbon in the surface snow of eastern Antarctica in 2011. J. Geophys. Res. 125, e2019JD030737. 10.1029/2019jd030737.

6. Oyabu, I., Kawamura, K., Fujita, S., Inoue, R., Motoyama, H., Fukui, K., Hirabayashi, M., Hoshina, Y., Kurita, N., Nakazawa, F., et al. (2022). Temporal variations of surface mass balance over the last 5000 years around Dome Fuji, Dronning Maud Land, East Antarctica. Clim. Past 19, 293–321. 10.5194/cp-19-293-2023.
7. Motoyama, H., Takahashi, A., Tanaka, Y., Shinbori, K., Miyahara, M., Yoshimoto, T., Fujii, Y., Furusaki, A., Azuma, N., Ozawa, Y., et al. (2021). Deep ice core drilling to a depth of 3035.22 m at Dome Fuji, Antarctica in 2001–07. Ann. Glaciol. 62(85–86), 212–222. 10.1017/aog.2020.84.

S2 Sample of source code

Code1 -GEE-

```
## This script is available in Google Earth Engine / Code Editor. Here, solar radiation data is extracted to make a chart. Please change "dataname" and "bandname" for any another variables. ##

// Sampling Locations
var p1 = ee.Geometry.Point([-59.120, 77.636]);
var p2 = ee.Geometry.Point([15.399, 78.229]);
var p3 = ee.Geometry.Point([39.583, -69.006]);
var p4 = ee.Geometry.Point([39.703, -77.316]);

var buffersize = 50e2;
var b1 = p1.buffer(buffersize);
var b2 = p2.buffer(buffersize);
var b3 = p3.buffer(buffersize);
var b4 = p4.buffer(buffersize);

var fc = new ee.FeatureCollection([
  ee.Feature(b1,{'name':'SIGMA-D'}),
  ee.Feature(b2,{'name':'SvalSat'}),
  ee.Feature(b3,{'name':'ShowaSta'}),
  ee.Feature(b4,{'name':'D-FujiSta'})
  ]);

// Temporal range
var date1 = '1950-01-01';
var date2 = '2050-01-01';

// ERA5-Land dataset
var dataname = "ECMWF/ERA5_LAND/MONTHLY";
var bandname = "surface_net_solar_radiation";
var  data  =  ee.ImageCollection(dataname).select(bandname).filterDate(date1,
date2);

// Quantity conversiont from energy ammount [J m-2] to mean flux [W m-2]
var Calc = function(image) {
  var output = ee.Image(0).expression(
    'M_SUM / (60*24*30)', {
      'M_SUM': image.select(bandname)
    });
  return image.addBands(output.rename('output'));
};
var data = data.map(Calc);

// Chart generation
var chart = ui.Chart.image.seriesByRegion({
  imageCollection: data.select('output'),
  regions: fc,
  reducer: ee.Reducer.mean(),
  scale: 500,
  seriesProperty: 'name',
  xProperty: 'system:time_start'
});
```

```
print(chart);

// Map visualization (selecting single snapshot)
var col = require('users/gena/packages:palettes').misc.tol_rainbow[7];
Map.addLayer(data.select('output').first(), {min: 0, max: 700, palette: col},
bandname);
Map.addLayer(fc,{'color': 'blue'}, 'Sampling Locations');
```

Code2 -Python-

```
## This script is optimized for use in Google Colab. Please use this script
coping and pasting to Google Colab. Dividing into multiple cells are
recommended at the positions of "#@title....". ##

#@title Installation, if needed
!pip install mido
!pip install dataclasses
!pip install google.colab
!pip install pandas
!pip install numpy

#@title Module reparation
import mido
from mido import Message, MidiFile, MidiTrack, MetaMessage
import dataclasses
from google.colab import drive, files
import pandas as pd
import numpy as np

#@title Access to a csv file in the google drive
drive.mount('/content/drive')
#@markdown Working directory and file
filepath = 'drive/MyDrive/' #@param {type:"string"}
filename = 'ee-chart.csv' #@param {type:"string"}

col= 'SIGMA-D'  #@param ["SIGMA-D", "SvalSat", "ShowaSta", "D-FujiSta"]
path = filepath + filename
data = pd.read_csv(path)
data = data[col]
# Prepare a list
line = np.array(data)

#@title Musical parameter setting
#@markdown Instrument (Default: 1)
inst = 1 #@param {type:"slider", min:1, max:128, step:1}
#@markdown Beat Per Minute
bpm = 80 #@param {type:"slider", min:40, max:200, step:1}
#@markdown Lowest tone (C5: 72)
#pch = 41 #@param {type:"slider", min:40, max:100, step:1}
pch = 72 #@param ["72", "60", "36"] {type:"raw"}
#@markdown Tone range (12= 8va)
tone_range = 24 #@param {type:"slider", min:12, max:72, step:1}
str_tone_range = str(tone_range)
#@markdown Basic Duration for one note
nv = 16 #@param ["2", "4", "8", "16", "32"] {type:"raw"}
```

S2 Sample of source code

```
#@title MIDI generation

# Make a MIDI file
mid = MidiFile()

# Make a track in the MIDI file
track = MidiTrack()
mid.tracks.append(track)
track.append(MetaMessage('set_tempo', tempo=mido.bpm2tempo(bpm)))
track.append(Message('program_change', program=inst, time=0))

# Define tone range
tone_mn = pch + 0
tone_mx = pch + tone_range
line_mn = np.nanmin(line)
line_mx = np.nanmax(line)
line = line * (tone_mx - tone_mn) / (line_mx - line_mn) + (tone_mx - line_mx
* (tone_mx - tone_mn) / (line_mx - line_mn))

# float-type data classified to be int as 12 tones
line = np.rint(line)

#Function definition
def tone(pch, nv):
    nval = int(480 * 4 / nv)
    track.append(Message('note_on', note=pch, velocity=64, time=0))
    track.append(Message('note_off', note=pch, velocity=64, time=nval))

# Apply the function to all values
for i in range(len(line)):
  x = i - 1
  judge = pd.isna(line[x])
  if judge == False:
    nn = int(line[x])
    tone(nn,nv)
#    print('tone')
  else:
    tone(25, nv)
#    print('na')

# File export
name = filename + '_' + str_tone_range + "_" +  col + '.mid'
mid.save(name)
files.download(name)
```

S3 Score

弦楽四重奏曲第１番

《極域エナジーバジェット》

String Quartet No.1

“Polar Energy Budget”

永井裕人

Hiroto NAGAI, Ph.D.

| Concert Note |

"Listen to the sound of the earth turning."－this is a quote of instructional art published by Yoko Ono in 1963. At first glance, this may be seen as an unrealizable instruction and a metaphor of some kind. In the present age, it is possible to observe the earth, collect digital data, convert it into audible feature, and listen to it.

Such process is called sonification. Familiar examples are heart rate monitors in hospitals and radiation dosimeters (Geiger counters). The National Aeronautics and Space Administration (NASA) has converted observation data from space-borne telescopes and planetary probes into sounds. Although it is now possible to listen to the data or information that could not be heard directly by people, can they really be called "music"? What is needed for the sonified parts to be incorporated into major music works? These questions are the motivations behind the creation of this work.

The materials for this work are originated from satellite-based earth observation data and climatic reanalysis datasets. They quantify the temporal changes of multiple physical quantities (i.e. shortwave and longwave radiation, surface temperature, cloud optical thickness, and precipitation). Four polar-regional points (i.e. an ice-core drilling site in the Greenland ice sheet, satellite communication facilities at Svalbard Islands, Showa Station in Antarctica, and Dome Fuji Station in Antarctica) are set for data sampling for two violins, a viola, and a cello. The composer's artificiality is added very little in the introduction chapter, whereas it appears more intensively in the latter chapter of the piece.

In meteorology and other earth science, we know that almost all atmospheric phenomena and ecosystem are fundamentally driven by the solar radiant energy. The solar energy transmitted in different forms causes the balance and order on the terrestrial surface, resulting in human’s civilization. Not only focusing on outreach against the global warming, but the composer here intends to express great possibilities to tell about numerous aspects of earth science with artistic expression supported by the sonification technique.

| Concert Note |

“Listen to the sound of the earth turning.”－これは 1963 年にオノ・ヨーコが発表したインストラクション・アートの言葉である。一見、実現できない指示であり、何らかのメタファーとも捉えられる。しかし様々なデジタル処理が可能になった現代では、地球を観測し数値データに直したものを音響や旋律に変換し、人間にも知覚可能な空気振動（＝音）として聴くことができるようになった。

このようなデータの可聴化はソニフィケーション（Sonification）と呼ばれる。身近には病院の心拍数モニターや放射線の線量計（ガイガーカウンター）が含まれ、米国航空宇宙局（NASA）は宇宙望遠鏡や惑星探査機の観測データを音に変換したものを公開している。直接に聞こえなかった響きを人間の可聴範囲に持ってくることはできるようになったが、果たしてそれが「音楽」と呼べるものであるのか、また広く「音楽」と呼ばれるためにはどのような作為を加えなければいけないのか、それが本作品を創作するに至った知的好奇心である。

本作品の素材は人工衛星などによる地球観測データについて、北極・南極周辺にある 4 地点の各種物理量の時間変化を数値化したものである。具体的には、短波および長波放射・地表面温度・雲による日射減衰率・降水量である。四重奏の高音パートから順に、グリーンランド氷床上・スバールバル諸島・南極昭和基地・南極ドームふじ基地の各地点が割り当てられ、楽曲後半になるほど作曲者の作為性が介入している。

気象学や地球科学では、ほぼ全ての気象現象やエコシステムが太陽の放射エネルギーを根源として成り立っていることを学ぶ。太陽からのエネルギーが形を変えながら伝わっていき、地球表層上のバランスと秩序、そして我々人間自身の営みを作り出している様を念頭に、音を編み込んでいった。「地球温暖化の警鐘を鳴らす」や「環境保護の重要性を訴える」という安直なテーマに限定せず、芸術表現としての可能性を広げることを重視する。

| Locations for sampling |

Violin 1st	[59.1°W, 78.6°N]	Greenland icesheet / SIGMA-D ice-core drilling site<br>グリーンランド氷床　SIGMA-D 氷床コア掘削サイト
Violine 2nd	[15.4°E, 78.2°N]	Svalbard islands / Satellite communication facilities<br>スバルバード諸島　衛星追跡管制局（SvalSat）
Viola	[39.6°E, 69.0°S]	Antarctica / Showa Station (NIPR)<br>南極　昭和基地
Cello	[39.7°E, 77.3°S]	Antarctica / Dome Fuji Station (NIPR)<br>南極　ドームふじ基地

| Input Parameters |

[Intro]	ERA5	Downward shortwave radiation, monthly mean（W/m^2）<br>地上月平均下向き短波放射量（W/m^2）
[A]	MODIS	Land surface temperature, 8-days mean（K）<br>地表面温度 8 日間平均値（K）
[B]-[C]	ERA5	Downward longwave radiation, monthly mean（W/m^2）<br>地上月平均下向き長波放射量（W/m^2）
[D]-[E]-[F]	Classic arrangements based on a motif (G-B-E-E) derived from the solar constant value (1.366 km/m^2) and top notes from Chapter [G] below.<br>太陽定数（1.366 kW/m^2）の音列（C-E-A-A）および後述[G]の先頭音列をモチーフとする古典的アレンジメント	
[G]	MODIS	Cloud optical thickness, monthly mean<br>月平均雲の光学的厚さ
[H]-[I]	ERA5	Precipitation, monthly mean（mm）<br>月平均降水量（mm）

ERA5： Climate reanalysis dataset provided by the European Centre for Medium-Range Weather Forecasts
欧州中期気象予報センター（ECMWF: European Centre for Medium-Range Weather Forecasts）が公開する気候再解析データセット

MODIS： An optical multispectral sensor, the Moderate-Resolution Imaging Spectroradiometer, onboard Terra/Aqua satellites (NASA)
Terra/Aqua 衛星（NASA）に搭載された中分解能スペクトル放射計（Moderate-Resolution Imaging Spectroradiometer）

| Acknowledgement |

This musical composition and performance were carried out in a research project financially supported by the Remote Sensing Technology Center of Japan. A climatic reanalysis dataset, ERA5, provided by the Copernicus Climate Change Service and MODIS datasets provided by Land Processes Distributed Active Archive Center (LP DAAC), NASA were imported for composition. Those contributions are greatly appreciated.

本作品は一般財団法人リモート・センシング技術センターによる 2022 年度 RESTEC 研究助成の支援を受け「衛星データ可聴化技法の探求～地球の響きと人間音楽の調和を探して～（研究代表者：永井裕人）」の研究の一環として創作されたものです。創作にあたり欧州 Copernicus Climate Change Service から提供される ERA5 気候再解析データセット、および米国 NASA Land Processes Distributed Active Archive Center (LP DAAC)から提供される MODIS データセットを使用しました。ここに謝意を表します。

Copyright © 2022 Hiroto NAGAI All rights reserved.

Score

弦楽四重奏曲第１番《極域エナジーバジェット》

永井裕人

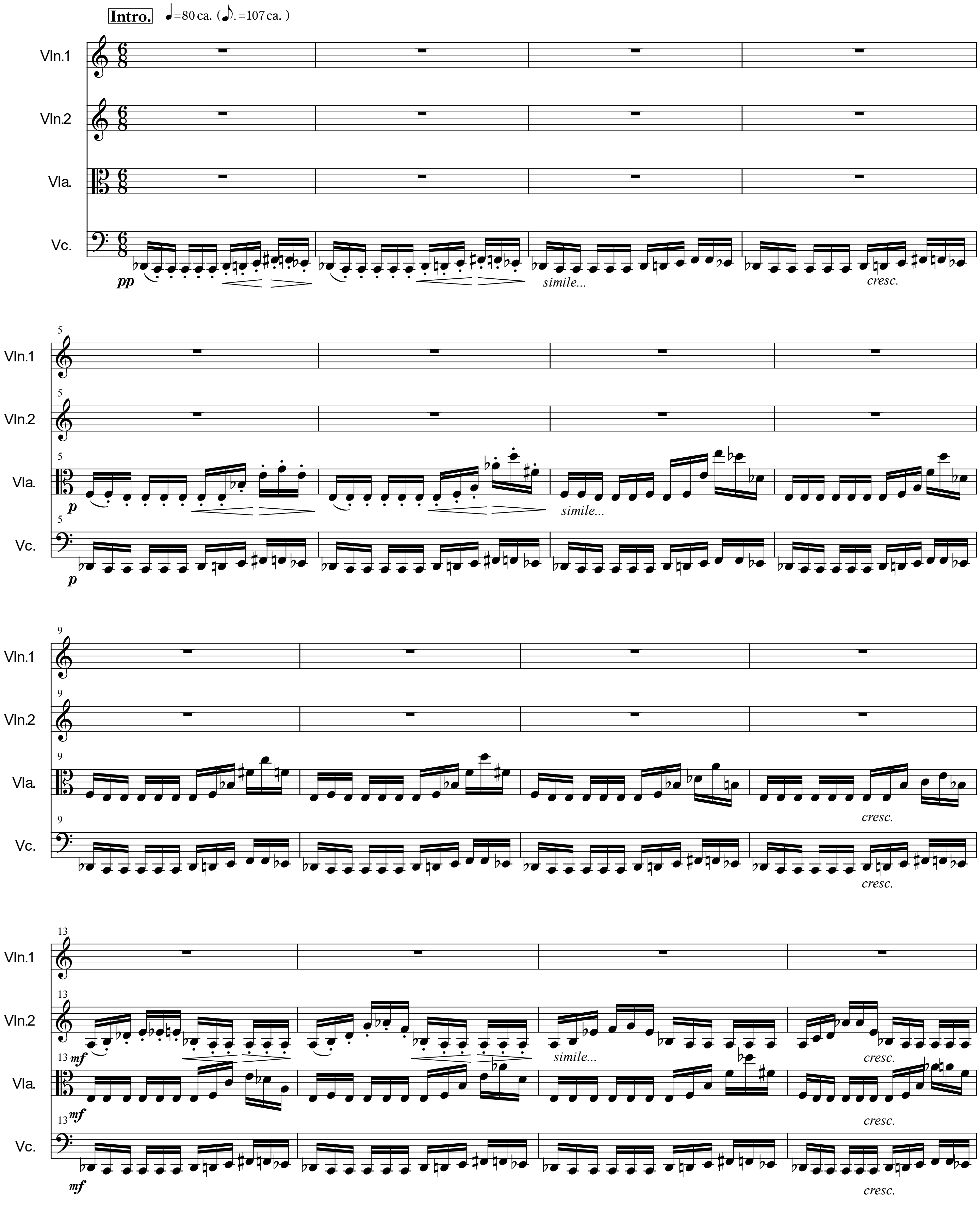

Copyright (c) 2022 Hiroto NAGAI All rights researved.

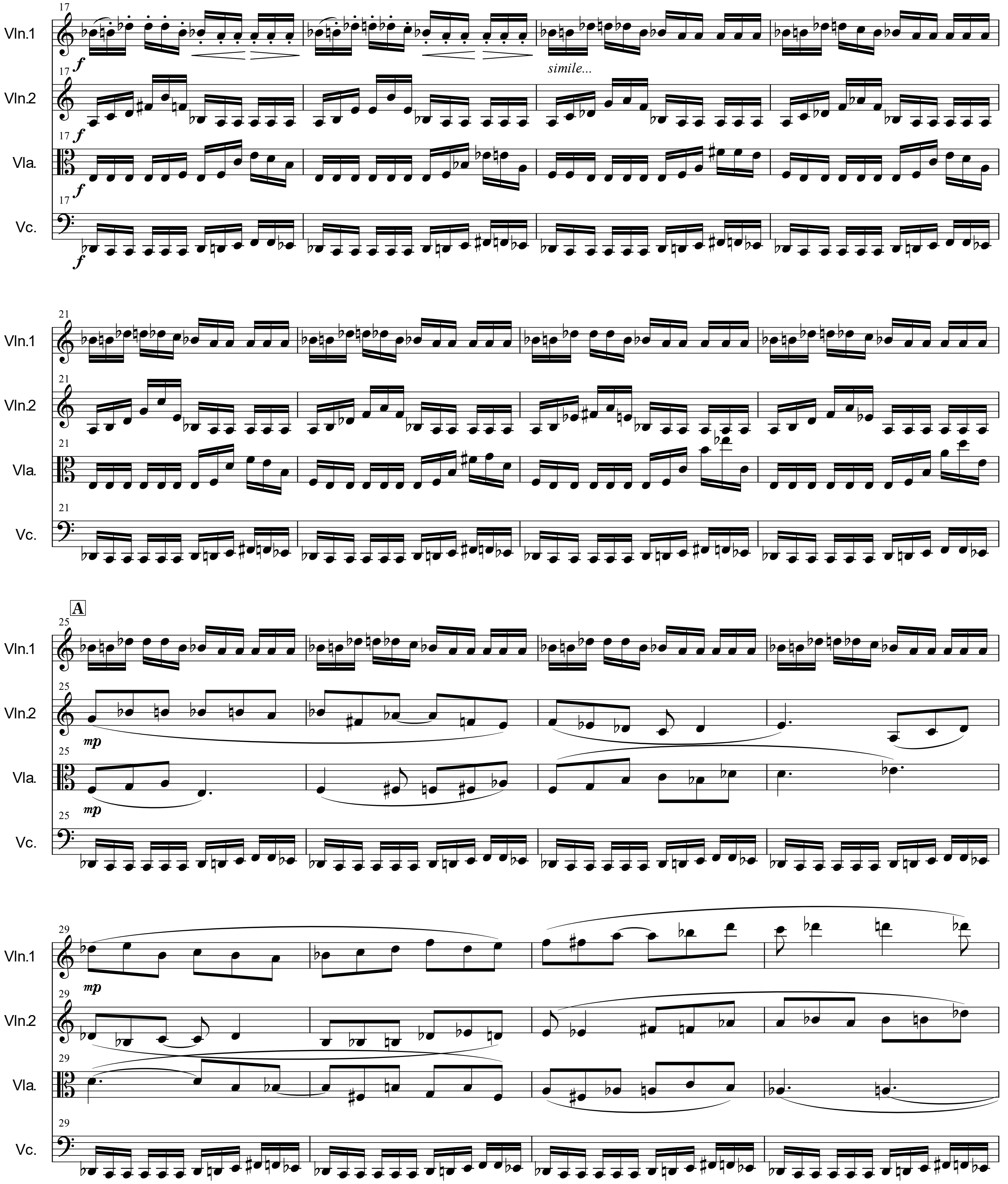
Vln.1
Vln.2
Vla.
Vc.
17
f
simile...
21
A
25
mp
29

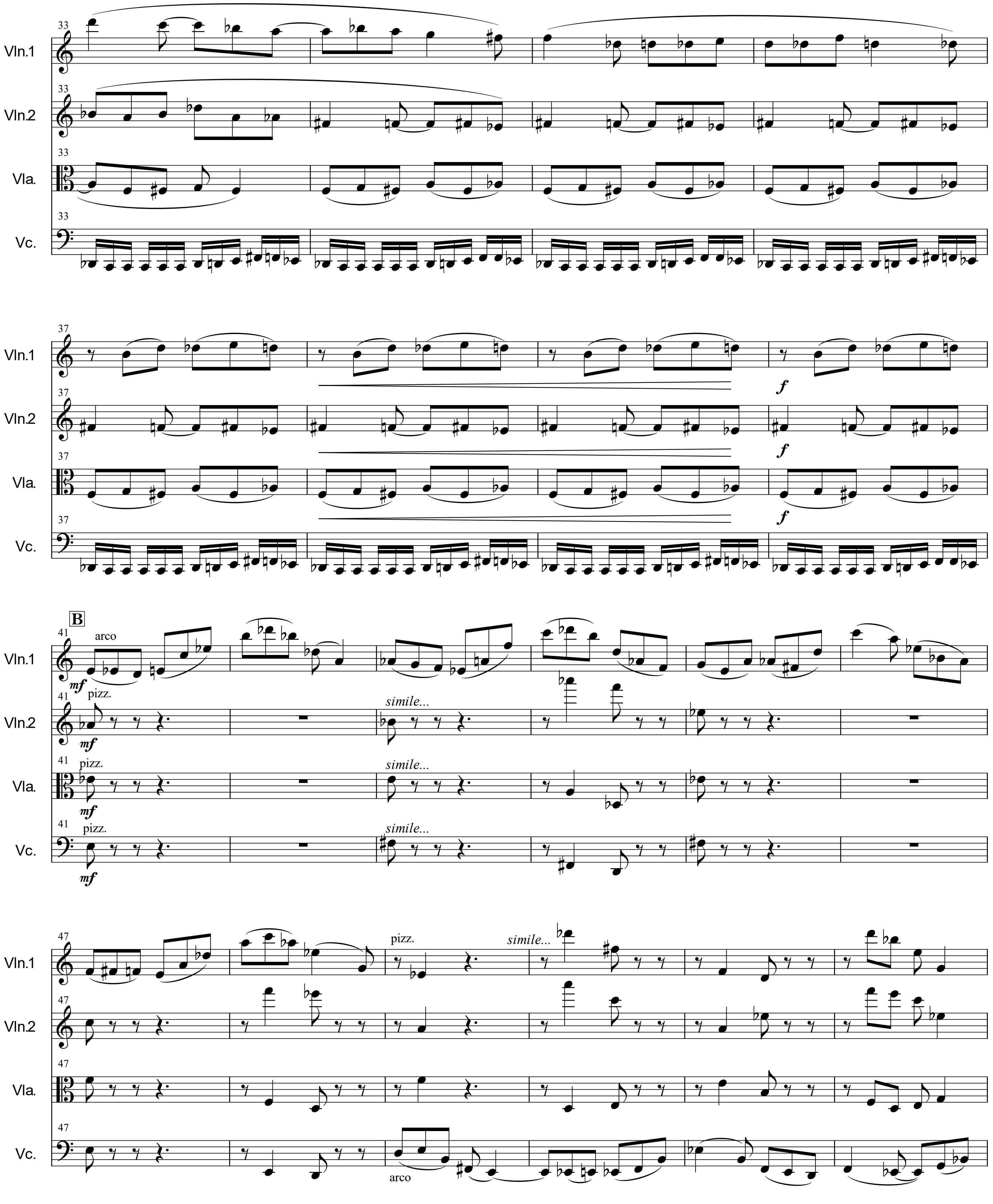
Vln.1
Vln.2
Vla.
Vc.
B
arco
pizz.
simile...
mf
f

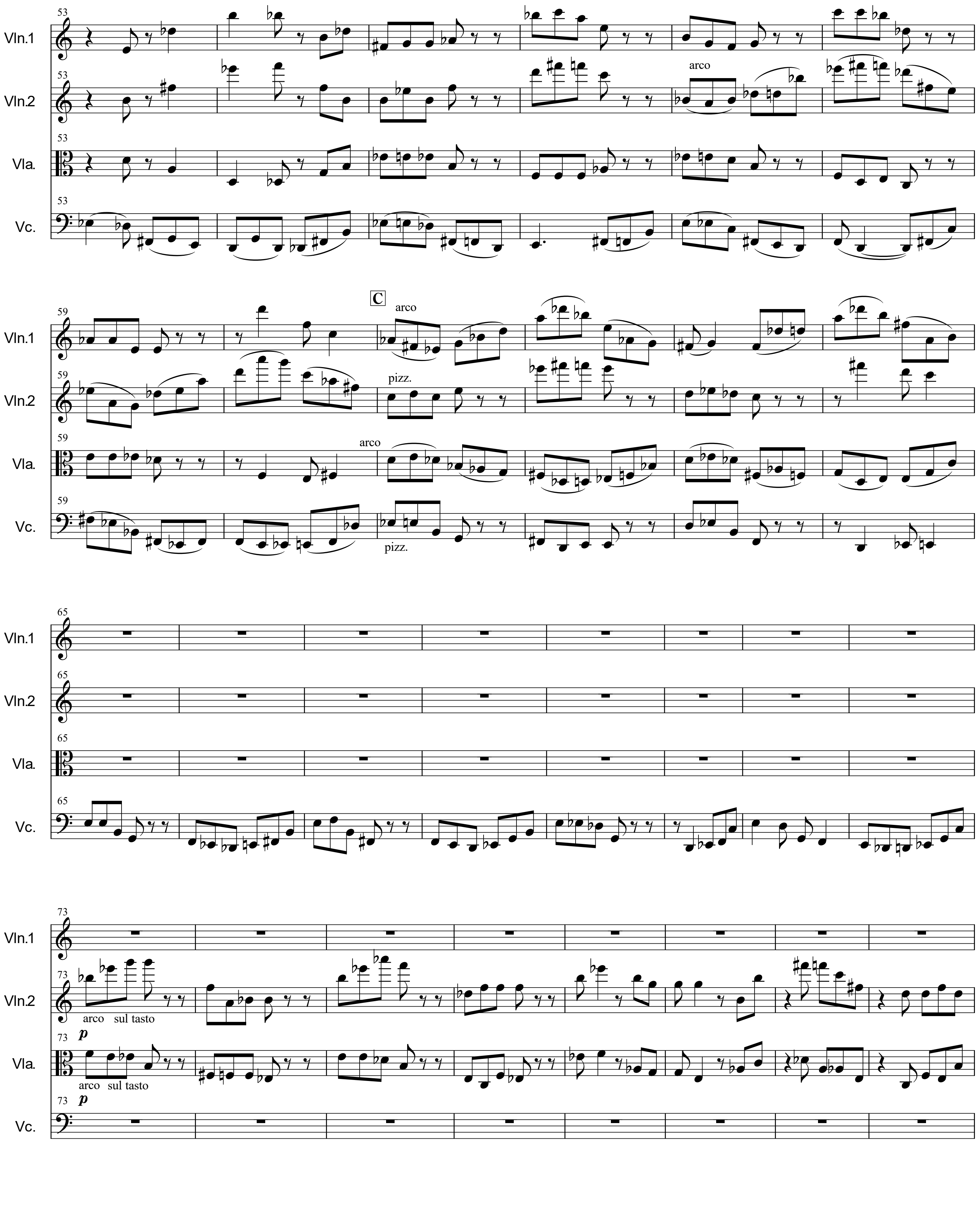
53
Vln.1
Vln.2
Vla.
Vc.
arco
59
C
arco
pizz.
arco
pizz.
65
73
arco sul tasto
p
arco sul tasto
p

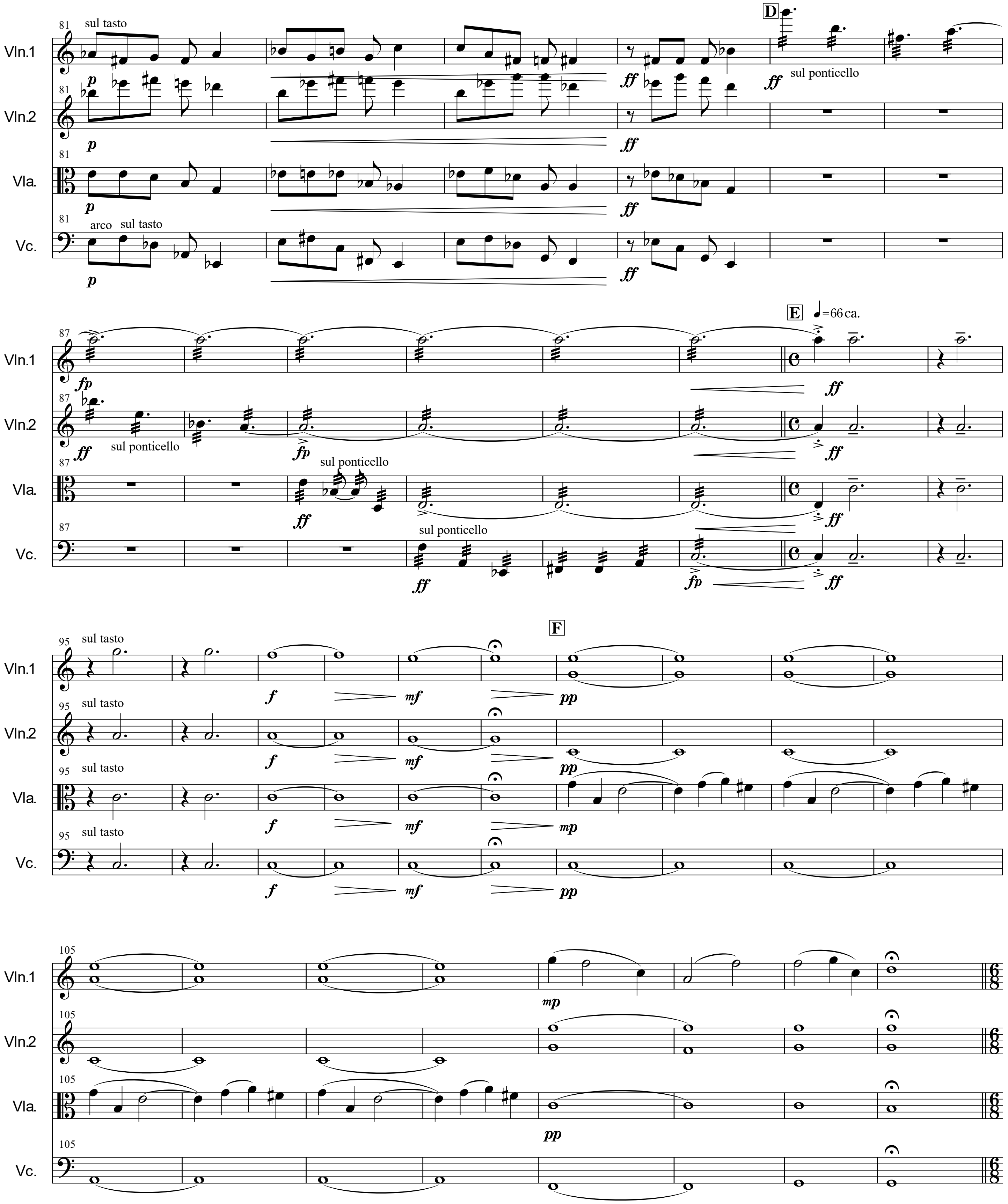
D
E
F
Vln.1
Vln.2
Vla.
Vc.
sul tasto
arco
sul ponticello
♩=66 ca.
p
ff
fp
f
mf
mp
pp

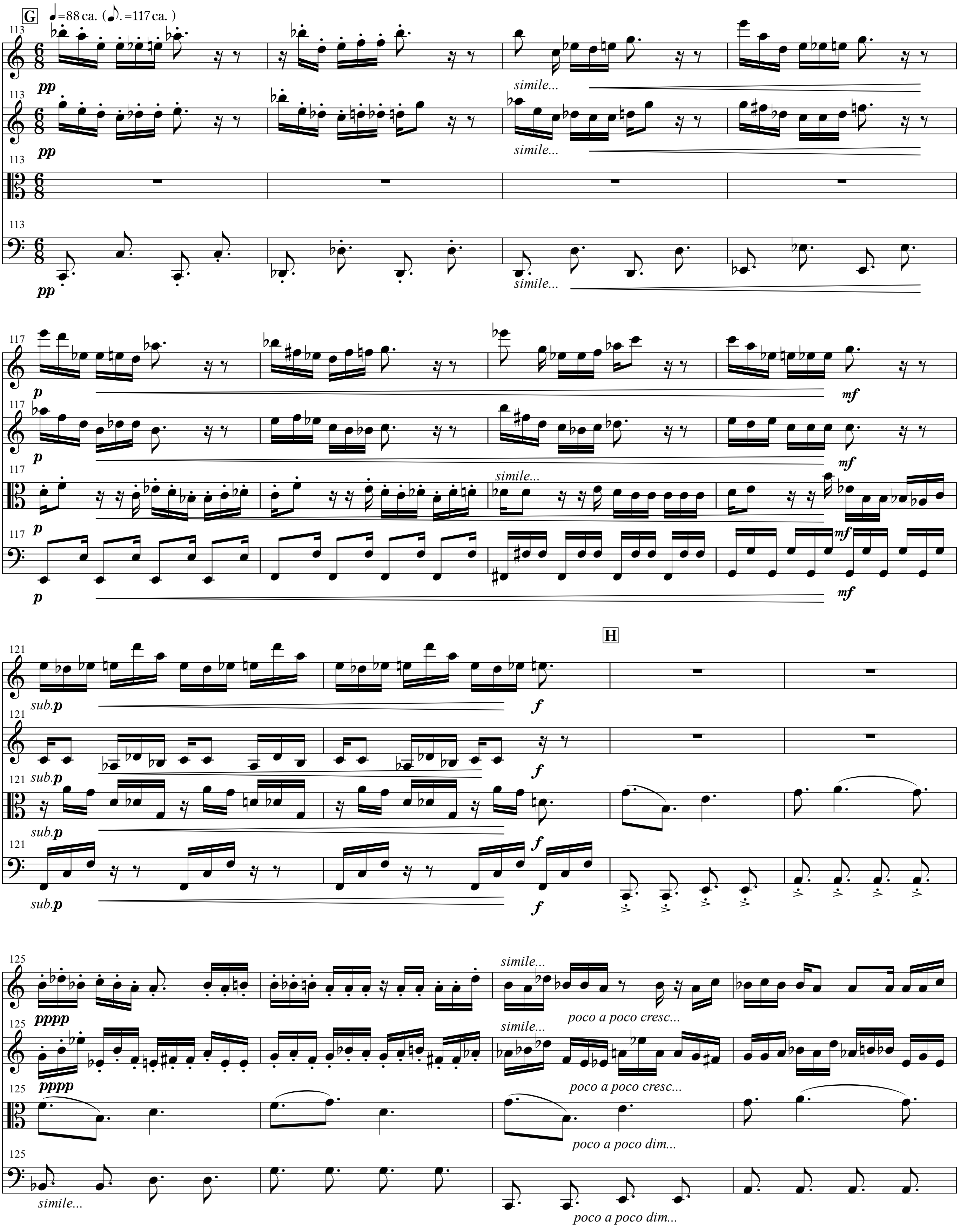
G
♩=88 ca. (♪. =117 ca.)
Vln.1
Vln.2
Vla.
Vc.
pp
simile...
p
mf
sub.p
f
H
pppp
poco a poco cresc...
poco a poco dim...

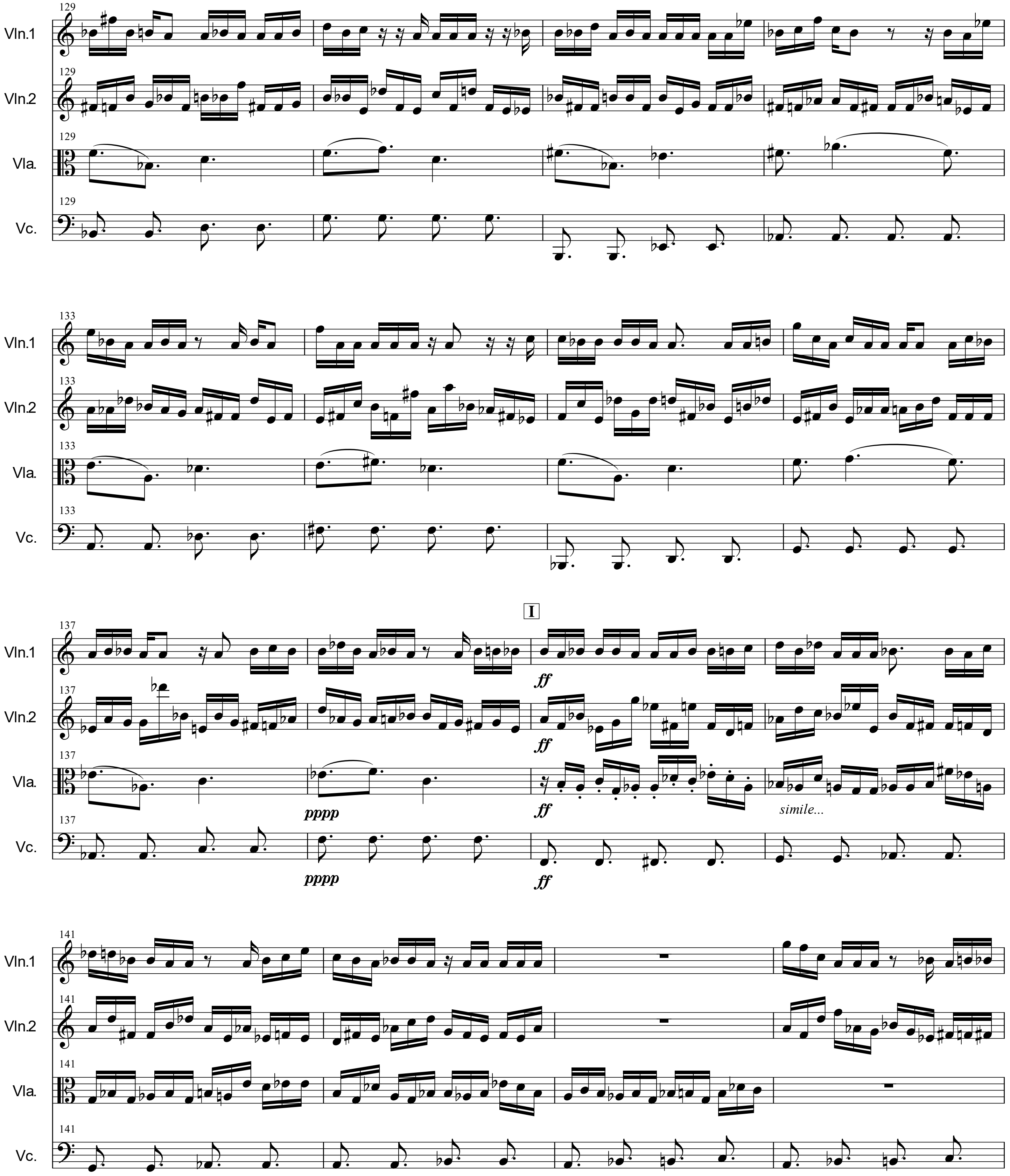
Vln.1
Vln.2
Vla.
Vc.
I
ff
pppp
simile...

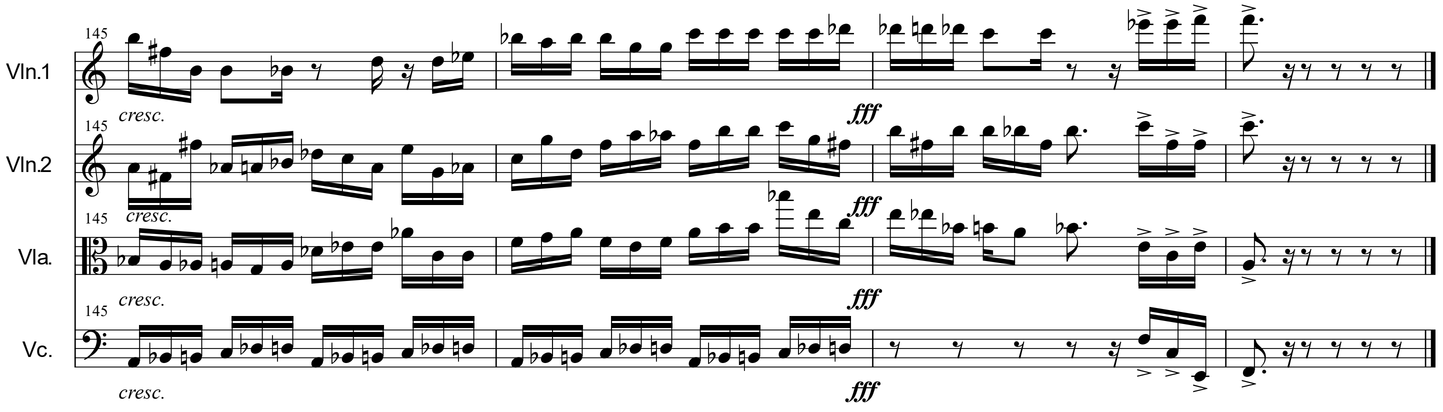
145
Vln.1
Vln.2
Vla.
Vc.
cresc.
fff

Vln.1

弦楽四重奏曲第１番《極域エナジーバジェット》

永井裕人

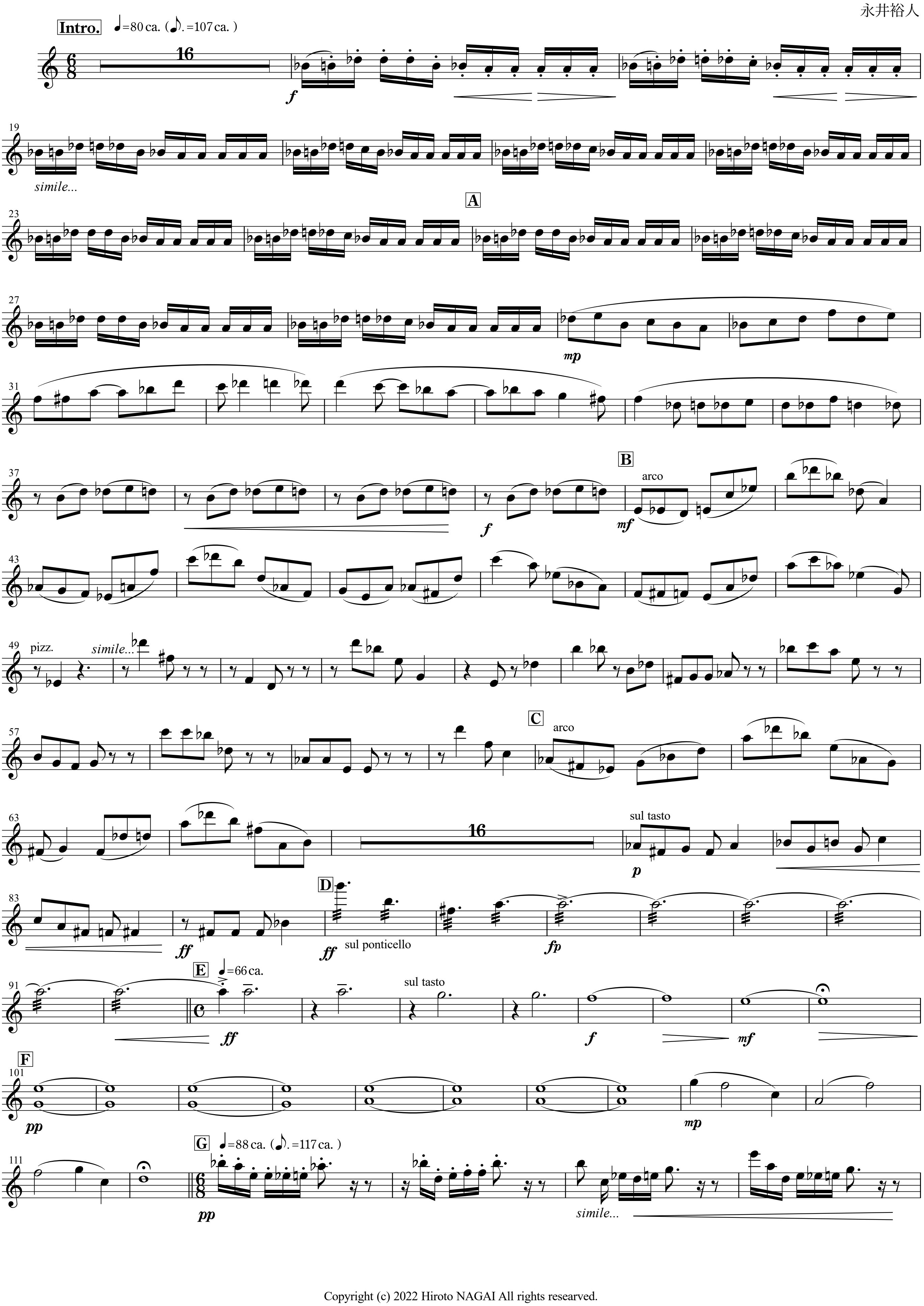

Copyright (c) 2022 Hiroto NAGAI All rights researved.

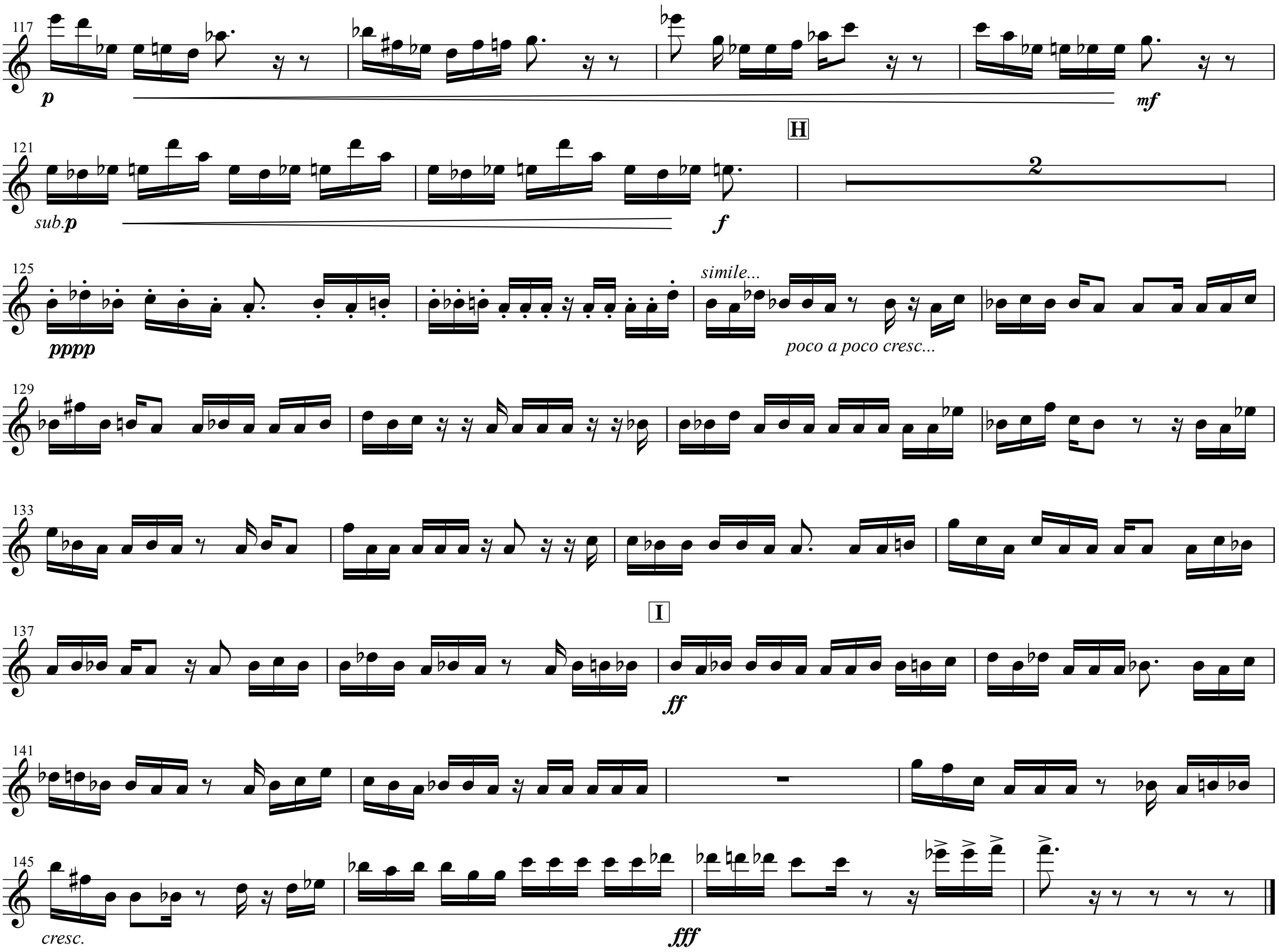
117
p
mf
121
sub.p
f
H
2
125
pppp
simile...
poco a poco cresc...
129
133
137
I
ff
141
145
cresc.
fff

Vln.2

弦楽四重奏曲第１番《極域エナジーバジェット》

永井裕人

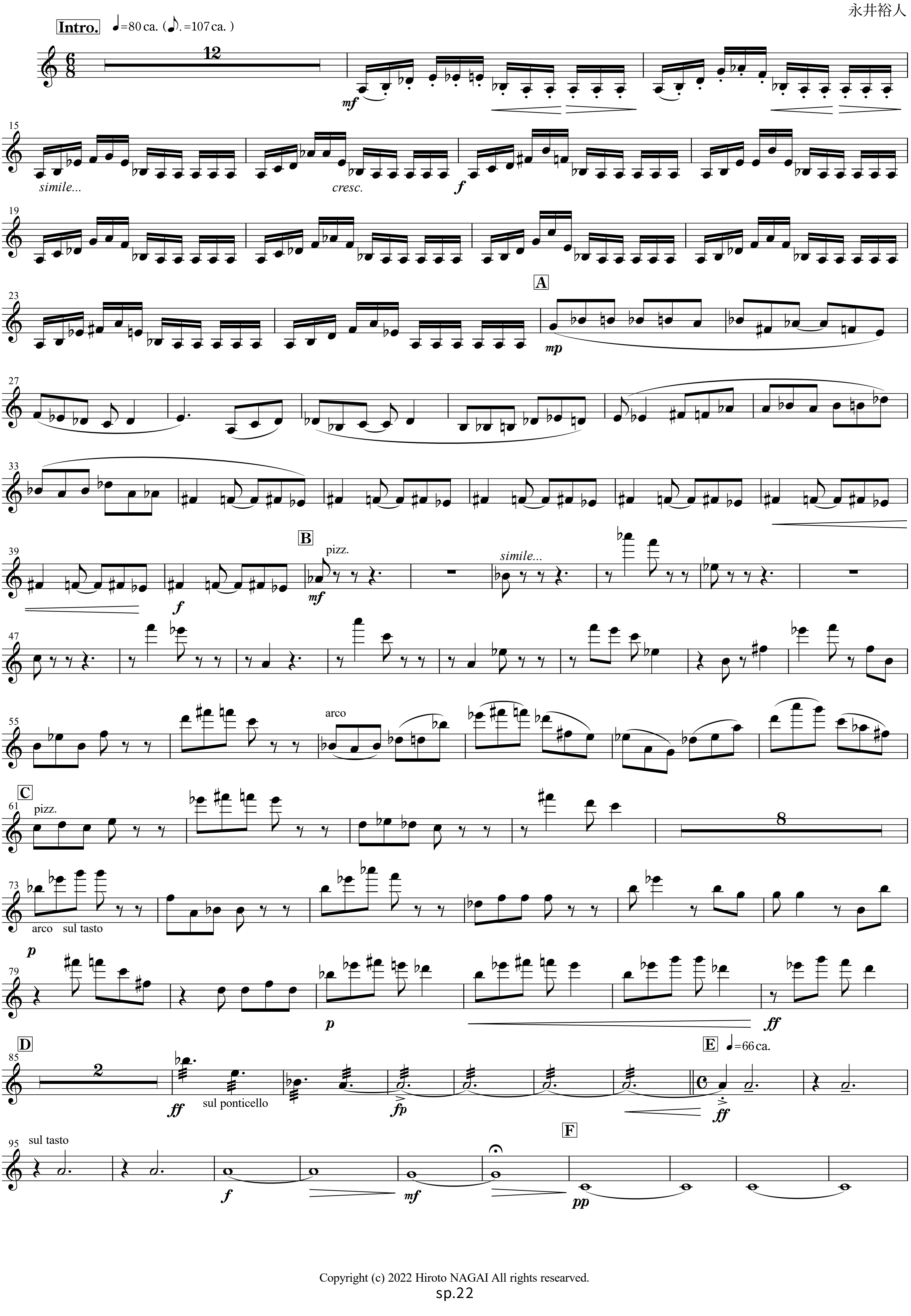

Copyright (c) 2022 Hiroto NAGAI All rights researved.

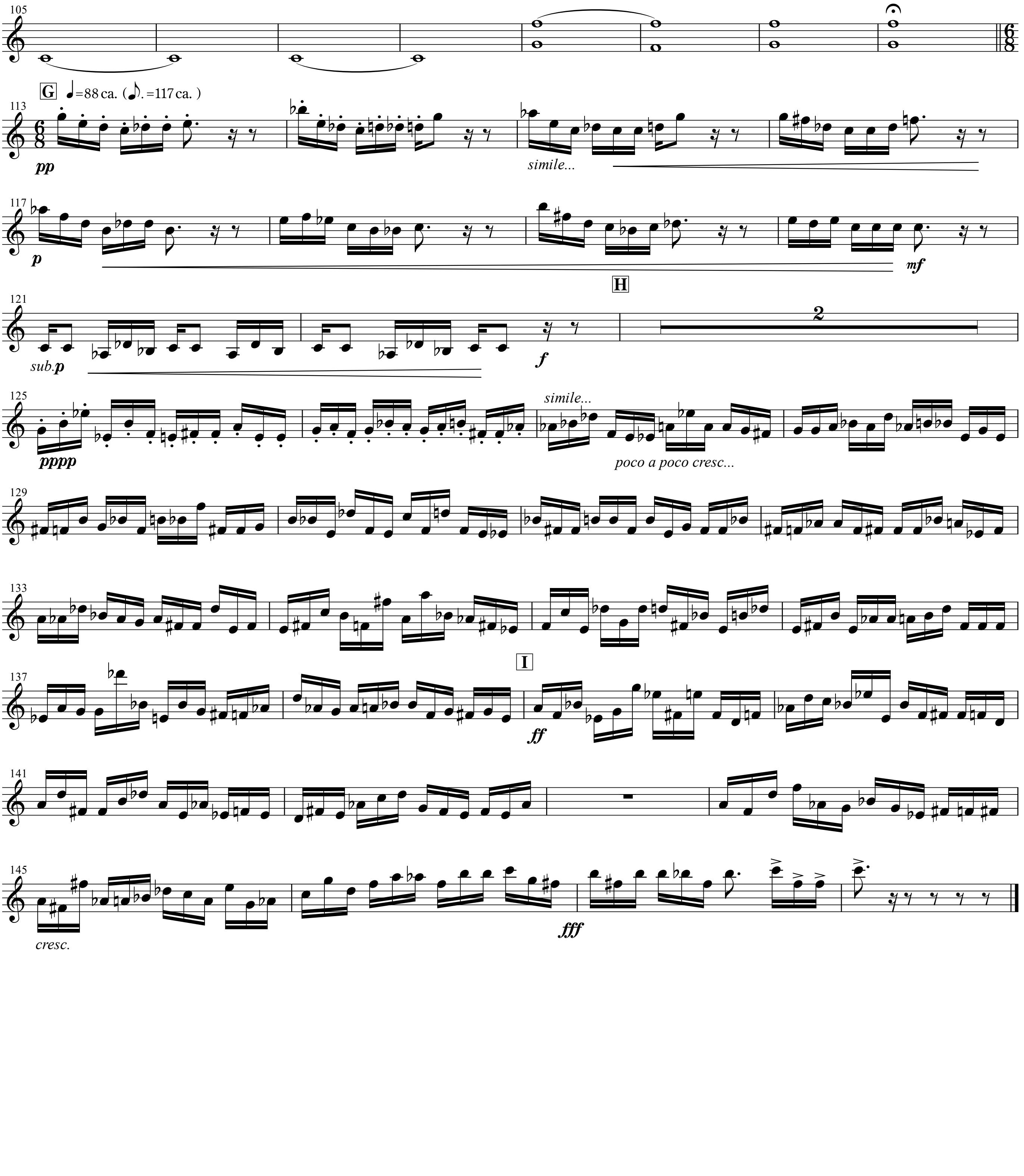
105
G
♩=88 ca. (♪. =117 ca.)
113
pp
simile...
117
p
mf
H
121
sub.p
f
2
125
pppp
simile...
poco a poco cresc...
129
133
137
I
ff
141
145
cresc.
fff

Vla.

弦楽四重奏曲第１番《極域エナジーバジェット》

永井裕人

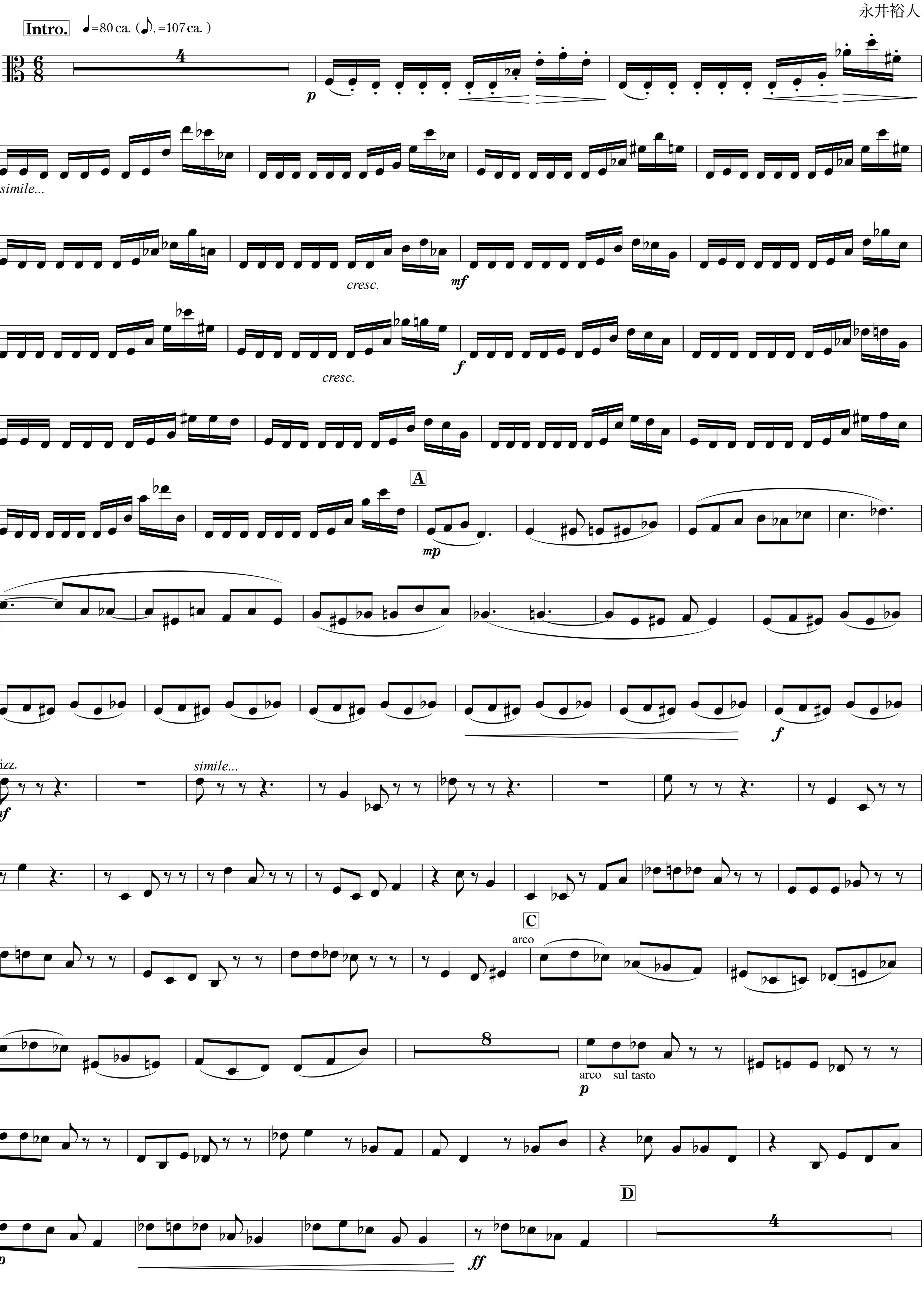

Copyright (c) 2022 Hiroto NAGAI All rights researved.

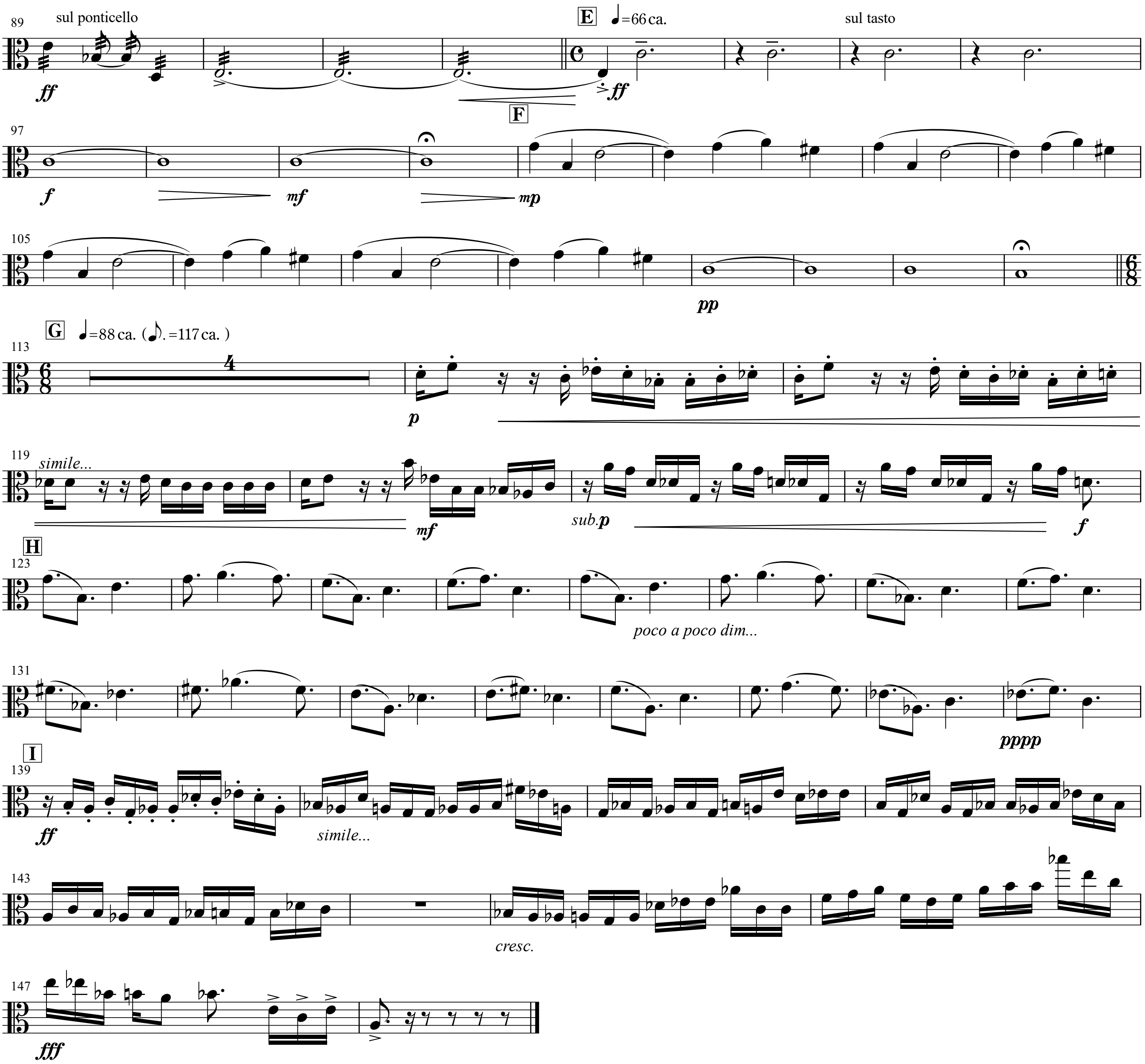
sul ponticello
E
=66ca.
sul tasto
F
G
=88 ca. (=117ca.)
simile...
sub.p
H
poco a poco dim...
I
simile...
cresc.

Vc.

弦楽四重奏曲第１番《極域エナジーバジェット》

永井裕人

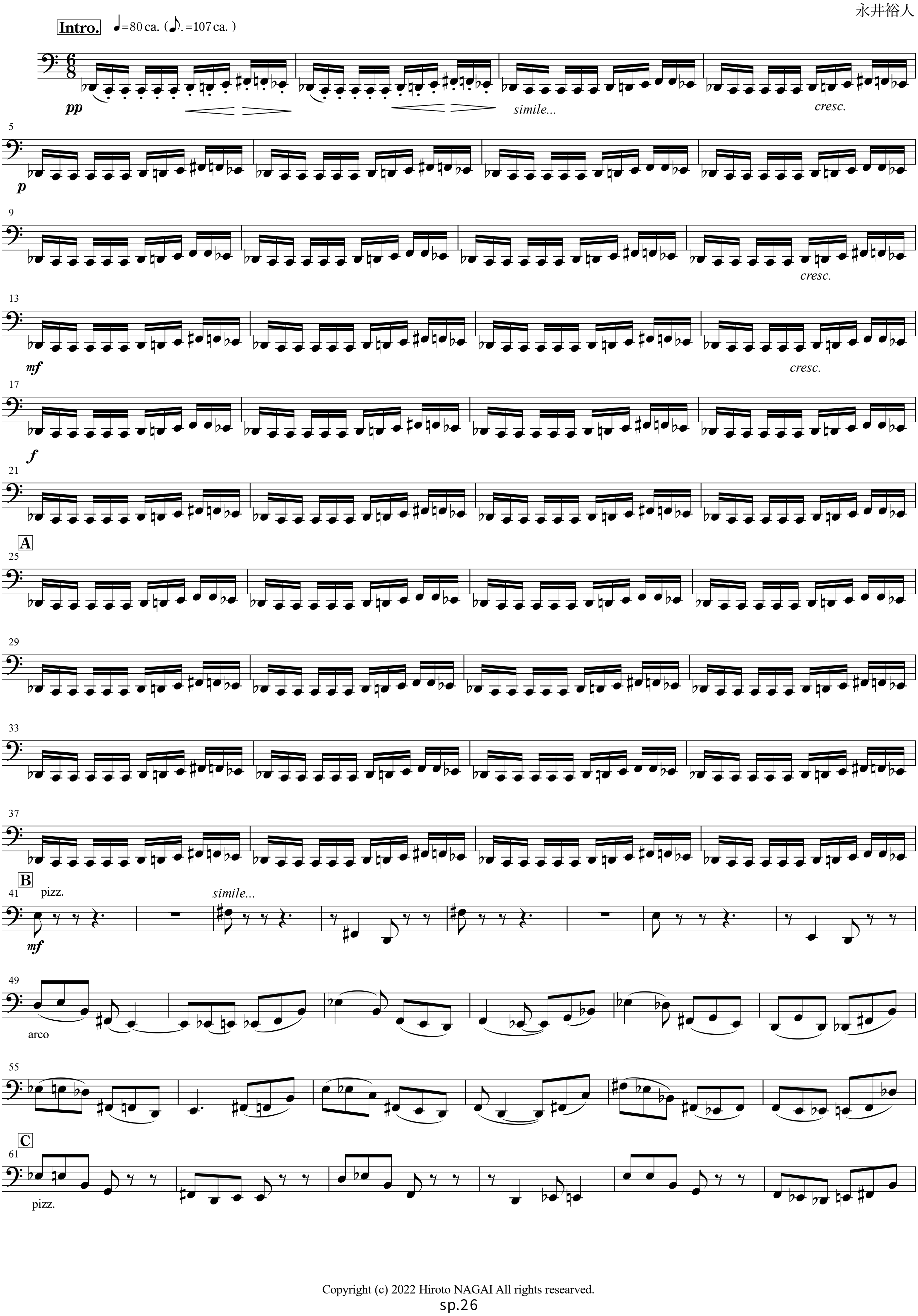

Copyright (c) 2022 Hiroto NAGAI All rights researved.

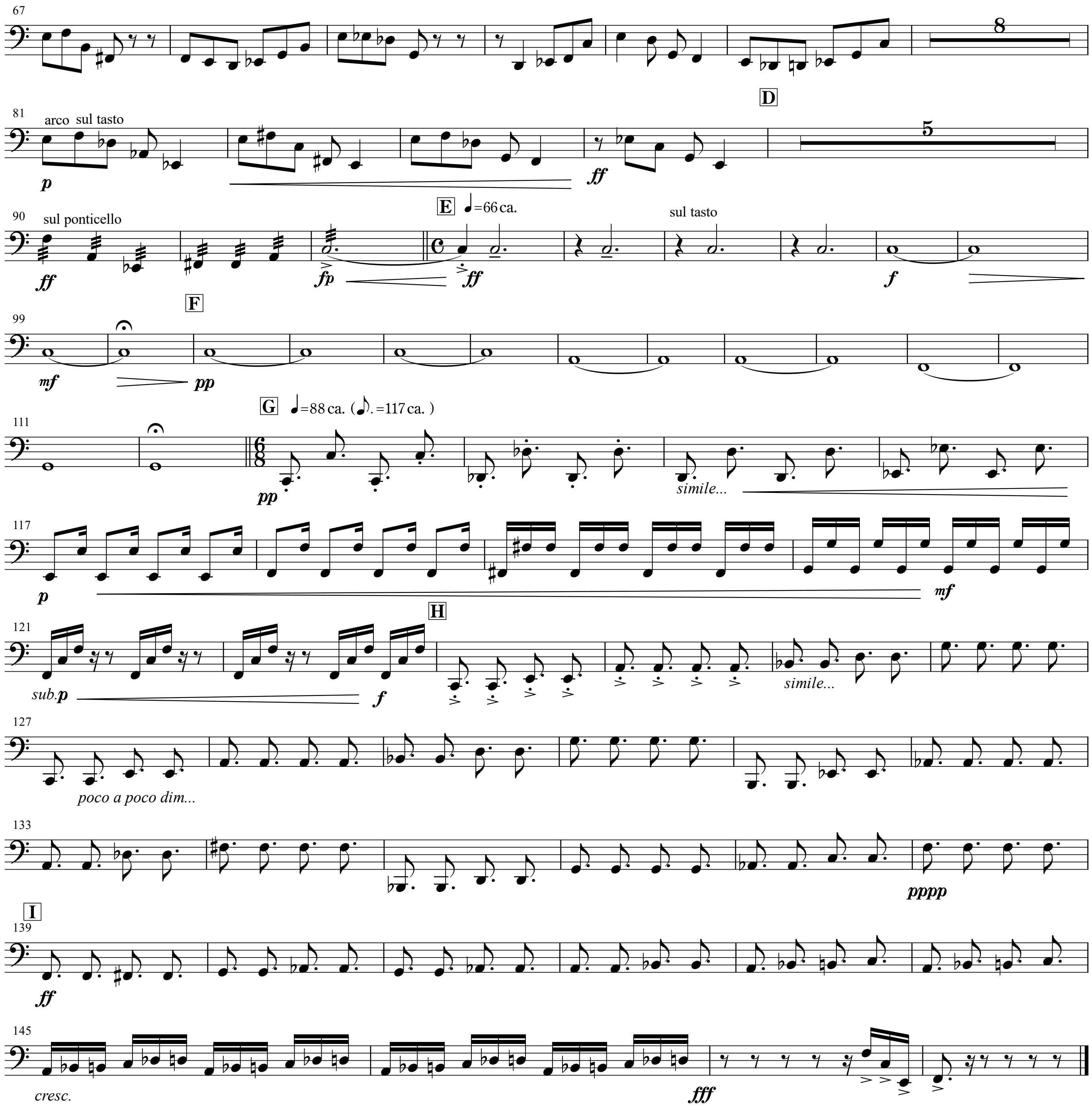
67
81
arco sul tasto
D
90
sul ponticello
E
=66 ca.
sul tasto
99
F
111
G
=88 ca. (=117 ca.)
simile...
117
121
sub.p
H
simile...
127
poco a poco dim...
133
I
139
145
cresc.

S4 Extracted script from the panel discussion

Player

HS: Haruka Sakuma The professional violinist who played the 2nd violin

Panelist

MO: Masato Ohki, Ph.D. A scientist of remote sensing in the Japan Aerospace Exploration Agency (JAXA)

AY: Aya Yamamoto, Ph.D. The director of corporate planning in a Japanese company, RESTEC, who process, analyze, and provide data from Earth observation satellites

MS: Momoko Sato A fouth-grade undergraduate student supervised by HN

TS: Tomohiro Sugaya A photographer, a practitioner of Japanese calligraphy, working for a Japanese IT company, Sakura internet Inc., operating a satellite data platform, “Tellus”

Moderator

HN: Hiroto Nagai, Ph.D. The composer, associate professor studying remote sensing and earth science

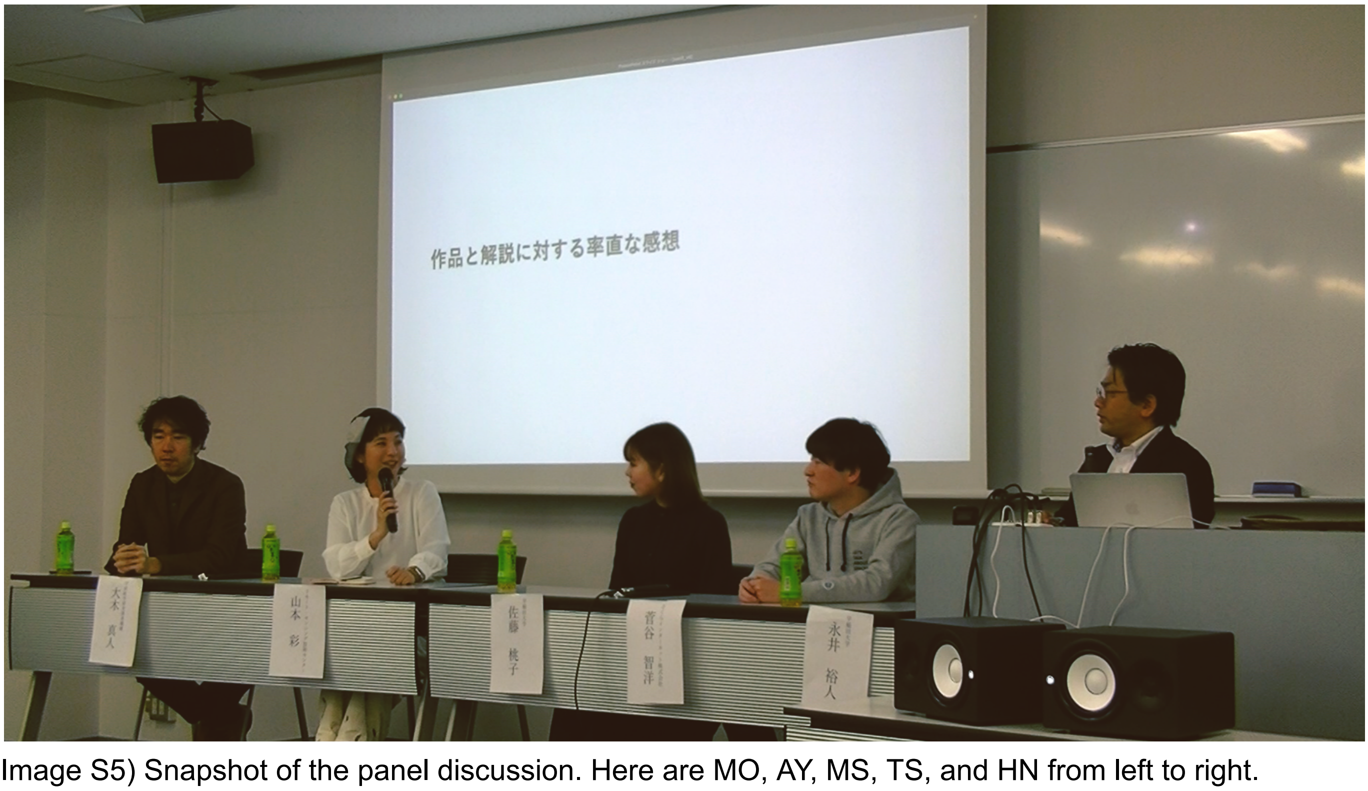

Image S5) Snapshot of the panel discussion. Here are MO, AY, MS, TS, and HN from left to right.

HN:

We had a performance of a string quartet with four people, and among them, the second violinist is here today, so I'd like to introduce her. This is Haruka Sakuma. We were too busy at the recording site to have a proper conversation, so please share your overall impressions after playing the piece.

HS:

Upon listening, my initial reaction was like, "What is this?" It felt like a typical contemporary piece. The flow of the music was a bit hard to memorize quickly, and it was quite challenging at first.

HN:

My impression was that all of you in the quartet catch on very quickly. Did you feel any discomfort in the music created from data?

HS:

As long as we have the sheet music, we just give it a try. Curiosity and interest took the lead.

HN:

I speculated that perhaps being deeply involved in music and the arts made the acceptance quicker. That was something I was pleased about. Thank you very much.

HN:

Please share brief impressions in order, something that can be a starting point for our discussion.

MO:

Today was my first time hearing the piece in this setting. I initially thought it would involve a rigorous, mathematical algorithm directly converting satellite data into sound. However, it turned out to be quite human and filled with personality, which was a bit surprising and interesting. The involvement of humans adds an intriguing element to the music. It's not a direct correspondence of data to sound. I imagine Mr. Nagai must have had specific intentions in choosing the four points in the polar region. Also, during the performance, the musicians were given the sheet music and had moments of hesitation or difficulty, like any random occurrences, which I think Mr. Nagai might have intentionally incorporated. The intriguing aspect of human touch was present.

Given that Mr. Nagai's main profession is as a researcher in the sciences, where he usually writes papers, it's challenging to bring out such human aspects. In the scientific world, reproducibility is essential. If, for instance, using data from a different location results in a completely different outcome, it undermines reproducibility, which is not accepted in the world of scientific papers. Art, on the other hand, values such uniqueness and human touch, which I found satisfying.

HN:

Yes, thank you. It hits the mark; there are moments when I wish I could write my papers a bit more freely. But, of course, it's a world where that's not possible. Composition is a contrasting activity.

AY:

When I heard just the intro in advance, I honestly wondered where this would go. It could be described as resembling Toru Takemitsu, but I questioned whether it truly qualifies as music. However, after hearing it today, I genuinely feel it has become something meaningful as music. There will likely be more discussions later, that it's also possible to make the composition method itself unique. Additionally, as mentioned earlier, I was listening while considering how much a scientist can do and where human intervention is acceptable. Thank you.

HN:

Thank you. Deciding how much to intervene is indeed challenging. I've been struggling with it. This time, I took the approach of gradually adding my own touch.

MS:

Initially, I saw the sheet music before hearing the music itself. At first, it seemed like the data would directly become sound, but then his touch was added. Deliberately removing certain sounds, creating rhythm, it transformed into something generally imagined as music. I thought about how much manipulation is needed for it to become music, for the sound to be listener-friendly. It's a piece with many considerations in that regard. I've mostly listened to classical music from the Romantic and Classical periods, so if there's going to be new music different from what we've had so far, it might start from aspects like these.

HN:

Thank you. Have you ever played piano music that sounds like this?

MS:

Not something this mechanical. Even in the genre of contemporary classical music, composers have all studied Classical, Romantic, and Baroque periods, so it doesn't sound as challenging as his work. The pieces I have played so far progressed beyond typical etudes like Sonatinas, but they didn't have much unexpectedness beyond losing tonality.

HN:

Thank you for your candid opinion.

TS:

When I first heard it, even though it was mentioned in the explanation, the subtle differences, removing sounds – I thought, performing this must be quite challenging. Listening to the piece, I did notice those subtle differences. As mentioned earlier, the choice of points and data selection can influence the outcome. It's something achievable through technology in this era. I got the impression of experiencing new music with various dimensions in the future.

HN:

That's delightful to hear. Thank you all for your individual perspectives.

= Presentaiton of related topics from the experts here =

HN:

What I'd like to delve into this time is the new possibilities in Earth science that can emerge through art. This is the vector where art brings benefits to science.

AY:

Earlier, Mr. Nagai composed a quartet based on data from four locations. In remote sensing analysis, you can typically visualize only about three layers like red, blue, and green. However, an orchestra can produce many more sounds, and humans can distinguish them. So, the idea occurred to me that by incorporating listening into the analysis method, we might be able to recognize even more information at once than through looking at images.

HN:

I chose a quartet because it's the simplest arrangement with four voices: soprano, alto, tenor, and bass. When I first started composing as a hobby, I used to layer many sounds, resulting in a chaotic, flat, and lacking sense of dimension. Bundling the movement of sounds is an essential point. By bundling them, it might be easier to find movements or changes in sound that stand out from the rest.

TS:

From the perspective of artistic creation, I have felt various things about the music this time. As Mr. Nagai mentioned earlier, there are discoveries that are different from visualization when it comes to making music. Beautiful color tones or beautiful scales are methodically established, but this time, I thought there might be infinite possibilities in how the music can be made beautiful based on the data or how the perception might change depending on the combination.

HN:

When forced to make judgments about which data to choose, I actually started having thoughts I've never had before. It revolves around whether the musical sequence can be considered beautiful or not. While I can determine whether a song I've created is beautiful, judging whether something from the natural world can be called beautiful is subjective and varies among

individuals. Now, let's move on to something related to Mr. Sato. For example, in fashion shows like Paris Collection or Milan Collection, there are many items created by cutting-edge designers that are not for everyday use. Mr. Sato worked part-time at a high-end boutique. Have you noticed or felt anything about the aesthetic consciousness of highly sensitive people in that cutting-edge fashion environment where celebrities visit and make expensive purchases?

MS:

Fashion shows are occasions to express the direction of a brand's concept, so the clothes presented are not meant for everyday wear. High-end boutiques have seemingly cute or peculiar items that become endearing once you become accustomed to them. Generally, even if you can buy a dress for ¥10,000, the store also has items priced at ¥300,000, which may be harder to accept. Similarly, this musical work may initially give off an impression of being difficult to listen to compared to classical or commercial music we've heard before. However, with familiarity, especially by explaining the story or meaning behind it, I believe it can be gradually embraced.

HN:

What one considers beautiful has certain rules, but it's a constantly changing element with time. Those on the cutting edge, like fashion shows I mentioned earlier, continue to change. So, people's aesthetic consciousness will continue to evolve. Beethoven's Symphony No. 5 was cutting-edge at the time, and now it's a familiar piece. I feel that creative activities will also evolve alongside the changing sensibilities of people. This project has just begun. I hope you will warmly watch over it in the future. Thank you all very much.
